# Gamma-delta T cells modulate the microbiota and fecal micro-RNAs to maintain mucosal tolerance

**DOI:** 10.1186/s40168-023-01478-1

**Published:** 2023-02-23

**Authors:** Rafael M. Rezende, Laura M. Cox, Thais G. Moreira, Shirong Liu, Selma Boulenouar, Fyonn Dhang, Danielle S. LeServe, Brenda N. Nakagaki, Juliana R. Lopes, Bruna K. Tatematsu, Luisa Lemos, Julia Mayrink, Eduardo L. C. Lobo, Lydia Guo, Marilia G. Oliveira, Chantal Kuhn, Howard L. Weiner

**Affiliations:** Ann Romney Center for Neurologic Diseases, Brigham and Women’s Hospital, Harvard Medical School, Boston, MA 02115 USA

## Abstract

**Background:**

Gamma-delta (γδ) T cells are a major cell population in the intestinal mucosa and are key mediators of mucosal tolerance and microbiota composition. Little is known about the mechanisms by which intestinal γδ T cells interact with the gut microbiota to maintain tolerance.

**Results:**

We found that antibiotic treatment impaired oral tolerance and depleted intestinal γδ T cells, suggesting that the gut microbiota is necessary to maintain γδ T cells. We also found that mice deficient for γδ T cells (γδ^−/−^) had an altered microbiota composition that led to small intestine (SI) immune dysregulation and impaired tolerance. Accordingly, colonizing WT mice with γδ^−/−^ microbiota resulted in SI immune dysregulation and loss of tolerance whereas colonizing γδ^−/−^ mice with WT microbiota normalized mucosal immune responses and restored mucosal tolerance. Moreover, we found that SI γδ T cells shaped the gut microbiota and regulated intestinal homeostasis by secreting the fecal micro-RNA let-7f. Importantly, oral administration of let-7f to γδ^−/−^ mice rescued mucosal tolerance by promoting the growth of the γδ^−/−^-microbiota-depleted microbe *Ruminococcus gnavus*.

**Conclusions:**

Taken together, we demonstrate that γδ T cell-selected microbiota is necessary and sufficient to promote mucosal tolerance, is mediated in part by γδ T cell secretion of fecal micro-RNAs, and is mechanistically linked to restoration of mucosal immune responses.

Video Abstract

**Supplementary Information:**

The online version contains supplementary material available at 10.1186/s40168-023-01478-1.

## Introduction

The intestine contains the largest amount of lymphoid tissue in the body and plays a critical role in developing a tolerogenic or inflammatory immune response to dietary and host antigens. There is a bi-directional interaction between intestinal immune cells and the gut microbiota to maintain homeostasis. γδ T cells play a major role in shaping the gut microbiota because of their innate-like and adaptive properties. γδ T cells that inhabit the intestinal epithelium are constitutively activated by commensal microbiota [1] and can rapidly respond to T cell receptor (TCR) signals in an MHC-independent manner [2] and to pattern recognition receptors signals including toll-like receptors (TLRs) [3] and NOD-like receptors (NLR) including NOD2 [4].

γδ T cells that reside in the gut lamina propria (LP) and intraepithelial lymphocyte (IEL) compartments maintain intestinal homeostasis by suppressing microbial populations through the secretion of cytokines such as IL-22 and IL-17 and growth factors including keratinocyte growth factor (KGF) that contribute to gut epithelial barrier integrity and induce the production of antimicrobial peptides (AMPs) by gut epithelial cells [5–8]. Intestinal γδ T cells also secrete AMPs including the regenerating islet-derived (Reg) protein family of C-type lectins, which protect against early invasion by intestinal-resident bacteria [9]. Consistent with this, mice deficient for γδ T cells (γδ^−/−^) have increased susceptibility to colitis [5, 10, 11].

In addition, we have identified and characterized a subtype of regulatory γδ T cells that express the latency-associated peptide (LAP; a membrane-bound TGF-β), which reside in the small intestine (SI) LP and Peyer’s patches and contribute to intestinal homeostasis by inducing regulatory (Treg) cells [12]. Thus, γδ T cell subtypes can have either pro-inflammatory or tolerogenic functions.

The role of γδ T cells in modulating the gut microbiota to maintain mucosal tolerance and intestinal homeostasis has become increasingly evident, but the mechanisms involved in this process remain elusive. While the microbial suppressive capacity of γδ T cells has been well established, whether γδ T cells positively select for beneficial microbes has not been investigated. Here we found that γδ T cells promote mucosal tolerance by shaping the gut microbiota, which in turn maintains small intestine γδ T cell populations. γδ^−/−^ mice developed an intestinal dysbiosis, which led to immune dysregulation characterized by increased Th17 and decreased Treg cells. This effect was associated with impaired migration of tolerogenic dendritic cells (DCs) to the mesenteric lymph node (mLN), and with decreased IL-10 production by CX3CR1^+^ mononuclear phagocytes (MNPs). Furthermore, we found that SI γδ T cells produced the miRNA let-7f which in turn promoted the growth of *Ruminococcus gnavus*, a Gram-positive anaerobic mucus-degrading-bacterial species. Colonization of γδ^−/−^ mice with *R. gnavus* or oral administration of let-7f regulated immune cells in the gut and restored mucosal tolerance. Thus, our study identifies new mechanisms by which intestinal γδ T cells modulate the microbiota via fecal miRNAs and play a critical role in maintaining intestinal homeostasis.

## Methods

### Animals

All animal experiments were performed according to an IACUC-approved protocol. Male and female, 8–12-week-old, on a B6 genetic background mice were used in this study. C57BL/6J wild type (WT) and TCRδ^−/−^ (γδ^−/−^) mice were purchased from the Jackson Laboratory and housed in a conventional specific pathogen-free facility at the Hale Building for Transformative Medicine, Brigham and Women’s Hospital, Harvard Medical School, and maintained on a 12-h light/dark cycle. WT and γδ^−/−^ mice were then bred to generate γδ^+/−^ heterozygous (HET) litters, which were then crossed to generate γδ^−/−^ mice, γδ^+/−^ (HET), and γδ^+/+^ (WT) littermate controls.

### Microbiota depletion and re-colonization

Microbiota was depleted by giving mice broad-spectrum antibiotics in their drinking water consisting of ampicillin, metronidazole, and neomycin at 1 mg/mL, and vancomycin at 0.5 mg/mL. To test the effect of microbiota depletion on oral tolerance, antibiotics were given for 3 days prior to and throughout the 5-day OVA feeding period, then antibiotics were replaced by water prior to immunization. To investigate the effect of antibiotics on immune populations, antibiotics were given for 5 days, and tissues were analyzed. For microbiota re-colonization experiments, 1 g of cecal contents was suspended in 15 mL of Pre-reduced Anaerobically Sterilized (PRAS) saline. Mice were treated with antibiotics for 3 days and switched back to water for 1 day, and then 200 μL of the microbiota suspensions were transferred to mice via oral gavage in a single dose. Three days later, OVA feeding for oral tolerance experiments commenced.

### Oral tolerance assessment

Oral tolerance was induced by continuously feeding animals with 8 mg/mL of OVA in the drinking water for 5 days. Control mice received only water. Three days after the last feeding, mice were immunized with 50 µg of OVA in complete Freund’s adjuvant (CFA) in the ventral flanks. In vitro recall responses were measured at day 10 after immunization. For this, splenocytes were stimulated with 4, 20, and 100 µg/mL of LPS-free OVA (antigen-specific stimulation) or 1 µg/mL of anti-CD3 (antigen non-specific stimulation as a control for proliferation), and proliferation was measured using ^3^H-thymidine incorporation. In some experiments, tolerance to OVA was measured by delayed type hypersensitivity (DTH) by injecting 60 µg of OVA into the hind paw and measuring foot pad swelling 1, 2, and 3 days later.

### Oral administration of let-7f

Mice were gavaged with let-7f or scrambled control sequence (1000 pmol/200 mL/mouse; Sigma-Aldrich) diluted in PBS, for 7 consecutive days. This dose was based on our previous study [13]. One day after the last dose of the miRNA, a group of mice were euthanized for flow cytometric analysis of the SILP, and another group was fed with OVA (as described above) for oral tolerance experiments.

### Bacterial colonization

*Ruminococcus gnavus*, ATCC 35913 Type Strain and *Parasutterella excrementihominis* DSMZ 21040, Type Strain were cultured on Brucella agar for 48 h, suspended in Pre-reduced Anaerobically Sterilized (PRAS) saline to an optical density of approximately 0.6. Two hundred milliliters of the bacterial suspensions was then transferred to mice via oral gavage 3 times in the first week, then once a week for 4 weeks. Oral tolerance and immunologic characterization were performed as described above.

### RT-qPCR

 αβ T cells, γδ T cells, and epithelial cells were sorted from the SILP (ab and gd T cells) and epithelial layer (αβ, γδ T cells, and epithelial cells) and lysed in RLT buffer (Qiagen). Lysate was centrifuged for 3 min at maximum speed at 4 °C and transferred to a genomic DNA eliminator column. RNA extraction was performed as per the manufacturer’s instructions (RNeasy Plus Micro Kit, Qiagen). Next, RNA was reverse-transcribed using the High-Capacity cDNA Reverse Transcription Kit with RNase Inhibitor (Thermo Fisher Scientific). Quantitative real-time PCR (RT-qPCR) was then performed on the cDNA using TaqMan Fast Universal PCR Master Mix (2X) no AmpErase UNG (Thermo Fisher Scientific) with a Vii 7 real-time PCR system (Applied Biosystems) with the following primers and probes: *Reg3a* (Mm01181787_m1), *Re3b* (Mm00440616_g1), and *Reg3g* (Mm00441127_m1). Quantitative PCR data were analyzed by the delta Ct method by normalizing the expression of each gene to *Gapdh* (Mm99999915_g1) or b-actin (Mm02619580_g1).

### Fecal microbe quantification by RT-qPCR

DNA in the mouse feces was extracted using a QIAamp Fast DNA Stool Mini Kit (Qiagen) and verified for specific bacteria abundance. RT-qPCR was conducted using a with a Vii 7 real-time PCR system (Applied Biosystems). *R. gnavus* and *A. muciniphila* were quantified by Taqman amplification reactions consisting of DNA, TaqMan Universal PCR Master Mix (Applied Biosystems), and primer pairs as follows: All bacteria (universal 16S rDNA, reference): Forward: TCCTACGGGAGGCAGCAGT, Reverse: GGACTACCAGGGTATCTAATCCTGTT, Probe: CGTATTACCGCGGCTGCTGGCAC [14]; *R. gnavus* 16S rRNA gene: Forward: CGCAGCAAACGCAATAAGTA, Reverse: CTGTCTCCTCTGTCCCGAAG, Probe: AAGCAACGCGAAGAACCTTA. *A. muciniphila* 16S rRNA gene: Forward: CGGTGGAGTAT GTGGCTTAAT, Reverse: CCATGCAGCACCTGTGTAA, Probe: CGCCTCCGAAGAGTCGCATG. The relative quantity was calculated using the comparative CT method normalizing to the amount of all bacteria in the sample [15].

### Flow cytometry

Small and large intestine lamina propria, intraepithelial lymphocytes (IELs), and mesenteric lymph node (MLN) were removed upon completion of the experiments and cells isolated for flow cytometric analyses. Dead cells were excluded based on 7-AAD (BD Biosciences) or the fixable viability dye Aqua Zombie (1:1000; Biolegend) staining.

For intracellular cytokine staining, cells were first stimulated for 3 h with PMA (phorbol 12-myristate 13-acetate; 50 ng/mL; Sigma-Aldrich) and ionomycin (1 μM; Sigma-Aldrich) and a protein-transport inhibitor containing monensin (1 mg/mL GolgiStop; BD Biosciences) before detection by staining with antibodies. Surface markers were stained for 25 min at 4°C in Mg^2+^ and Ca^2+^ free HBSS with 2% FCS, 0.4% EDTA (0.5 M), and 2.5% HEPES (1M), then were fixed in Cytoperm/Cytofix (eBioscience) and permeabilized with Perm/Wash Buffer (eBiosciences). Flow cytometric acquisition was performed on a Fortessa or Symphony A5 instruments (BD Biosciences) by using DIVA software (BD Biosciences), and data were analyzed with FlowJo software versions 10.1 (TreeStar Inc). Intracellular staining antibodies used are as follows: FITC-anti-Foxp3 (FJK-16s; 1:100; Thermo Fisher), BV421-anti-IFN-g (XMG1.2; 1:300; Biolegend), PE-Cy7-anti-IL-17A (eBio17B7; 1:300; eBioscience), PE-anti-IL-10 (JES5.16E3; 1:100; eBioscience). Other antibodies included the following: FITC-anti-CD45 (30-F11; 1:200; Biolegend), AF700-anti-CD45 (30-F11; 1:200; Biolegend), BV605-anti-CD11b (M1/70; 1:300; Biolegend), PE-anti-Vb5.1/5.2 (MR9-4; 1:100; Biolegend), BV421-anti-CD326 (G8.8; 1:100; Biolegend), BV711-anti-CD103 (2E7; 1:200; Biolegend), BV421-anti-XCR1 (ZET; 1:200; Biolegend), PE-Cy7-anti-Sirpα (P84; 1:300; Biolegend), PE-anti-CX3CR1 (SA011F11; 1:300; Biolegend), AF700-anti-CD3e (17A2; 1:300; Biolegend), BV605-anti-TCRgd (H57-597; 1:300; Biolegend) and BV421-anti-TCRgd (GL3; 1:200; Biolegend), PerCP-efluor710-anti-TCRgd (GL3; 1:200; Biolegend), PECy7-anti-CD4 (GK1.5; 1:400; Biolegend), BUV496-anti-CD4 (GK1.5; 1:400; BD Biosciences), BV711-anti-CD8a (53-6.7; 1:300; Biolegend), and APC-anti-CD8b (YTS156.7.7; 1:300; Biolegend).

### Microbial community analysis

Microbiota samples were collected and stored at −80°C. DNA was extracted using the PowerLyzer DNA Extraction Kit (Qiagen). To assess microbial composition, the microbial 16S rRNA gene was amplified with barcoded fusion primers (515F, 806R) targeting the V4 region as described [16] using HotMaster Mix (QuantaBio). Amplicons were quantified with Quant-iT PicoGreen dsDNA regent (LifeTechnologies) and pooled at equal DNA quantity. Excess primers were removed with the QiaQuick PCR purification kit (QIAGEN), and each pool of up to 96 samples was quantified with the Qubit high sensitivity dsDNA Assay (LifeTechnologies) and combined at equal concentration. The amplicons were sequenced on the Illumina MiSeq platform at the Harvard Medical School Biopolymer Facility, with 30% PhiX spiked into the amplicon pool to improve the signal from a low diversity library. Paired end reads were sequenced with 151 base pairs for the forward and reverse read and 12 bases for the barcode. Data was processed using the QIIME software following an established protocol [17]. Briefly, sequences were de-multiplexed and quality filtered in which reads were truncated if two consecutive bases fall below a quality score of Q20 (1% error), and reads that were < 75% of full length were discarded. Significant differences were determined by linear discriminant analysis effect size (LEfSe) [18], and predicted metagenomic profiles were constructed using Phylogenetic Investigation of Community by Reconstruction of Unobserved States (PICRUSt) [19].

### Nanostring

Small intestine gene expression was measured using the nCounter GX Mouse Immunology assay (NanoString Technologies, Seattle, WA). Differential testing was performed using the NSolver Advanced Analysis Module in which the distribution of each gene is used to select the optimal model for differential expression. Pathway Analysis was conducted using Ingenuity Pathway Analysis (Qiagen). Fecal miRNAs were measured using the nCounter mouse miRNA assay. Counts were normalized to a panel of housekeeping genes, and significant differences were detected by Student’s *t* test and statically relevant results consisted in *p* < 0.05.

### Histopathology

Small intestines (duodenum, jejunum, and ileum) from WT and γδ^−/−^ mice colonized or not with *R. gnavus* (gavaged once a week for 4 weeks) were removed, and 5-μm serial sections were stained with PAS for mucus analysis. Percentage of PAS per mm^2^ of tissue were calculated by measuring PAS^+^ vs. PAS^−^ area in 5 fields per sample slice using FIJI. A separate set of samples were fixed in PFA 4% for 24 h, transferred to a 30% sucrose solution for 48–72 h, and then embedded in OCT for sectioning. Slices of 20-μm serial sections were stained with AF-488-conjugated wheat germ agglutinin (WGA) and analyzed using a fluorescence microscope (DMi8, Leica).

### Statistics

GraphPad Prism 9.0 was used for statistical analysis (unpaired, two-tailed Student’s *t* test or one-way ANOVA, followed by Tukey multiple comparisons). Statistical analysis for 16S rRNA sequencing data is described above. Differences were considered statistically significant with a *p* value of less than 0.05.

## Results

### The microbiota promotes the induction of oral tolerance and maintains gd T cells

**Fig. 1:**
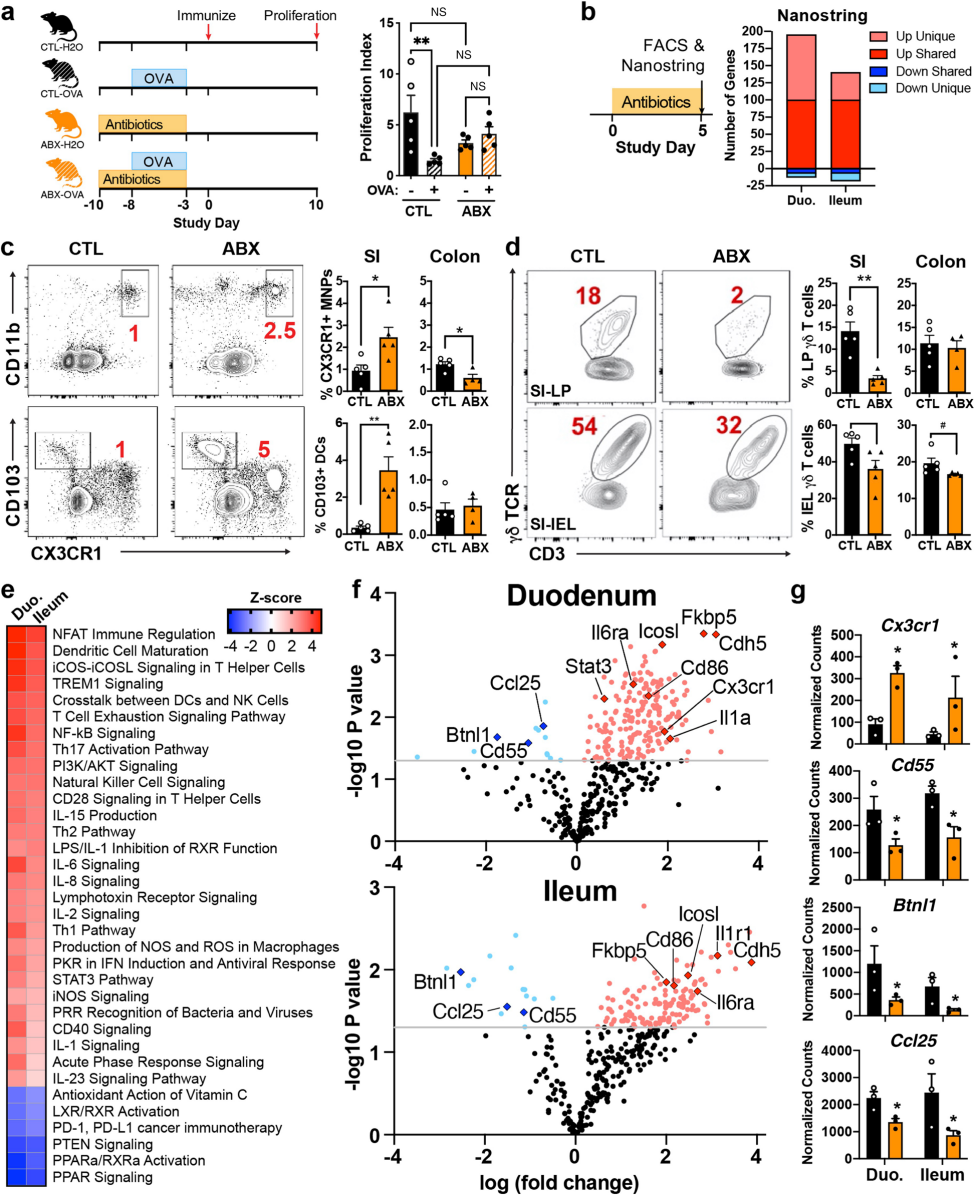
The microbiota is necessary for oral tolerance and maintains γδ T cell populations. **a** Microbiota was depleted with a combination of 4 antibiotics (ABX) in the drinking water for 3 days, and control mice did not receive antibiotics (*n* = 10 mice each). Half of each group were then fed OVA in the drinking water for 5 days and antibiotics were maintained during the OVA feeding period. Antibiotics and OVA were stopped and 2 days later mice were immunized with OVA/CFA. Responsiveness to OVA was measured by splenocyte proliferation upon 100 µg/mL of OVA stimulation. Data are mean ± SEM; *n*=4–5 mice/group; one-way ANOVA. **b** WT mice were treated with antibiotics for 5 days and on the day after, immunologic populations related to tolerance were measured by flow cytometry and transcriptional responses in the gut was measured by Nanostring. **c, d** FACS plots and bar graphs showing frequencies of live CD11c^−^CD11b^+^CD103^−^CX3CR1^+^ MNPs and CD11c^+^CD11b^−^CD103^+^CX3CR1^−^ DCs (**c**) and CD3^+^TCRγδ^+^ γδ T cells (**d**) from small intestine (SI) and colonic lamina propria (LP; **c, d**), intraepithelial layer (IEL, **d**) of control (CTL) or ABX-treated mice. Data are mean ± SEM; *n*=4–5 mice/group; Student’s *t* test. * *p* < 0.05, ** *p* < 0.01. **e, f** Transcriptional analysis of duodenum and ileal tissue of naïve antibiotic-treated mice measured by Nanostring NCounter Mouse Immunology Panel. **e** Canonical pathways altered by antibiotic treatment determined by Ingenuity Pathway Analysis. **f** Volcano plot of genes significantly altered in the duodenum and ileum (NCounter, advanced analysis module, *p* < 0.05). **g** Expression of selected genes in both the duodenum and ileum related to immunologic populations we observed in our flow cytometry data. * *p* < 0.05, NCounter Advanced Analysis Module. Results are representative of at least two independent experiments

In health, the gut microbiome constantly interacts with immune cells to maintain intestinal tolerance and homeostasis. In this sense, oral tolerance plays a critical role in maintaining the gut homeostatic environment to prevent intestinal inflammation in response to ingested antigens [20]. To investigate how the gut microbiota shapes immune responses necessary for oral tolerance, we depleted the microbiota during the time of oral tolerance induction. Control mice fed OVA showed intact induction of oral tolerance as evidenced by decreased proliferation and reduced footpad swelling when challenged with OVA (Fig. 1a; Supplemental Fig. 1a, b). Treatment with quadruple antibiotics (ampicillin, metronidazole, neomycin, and vancomycin) 3 days prior to and throughout the OVA 5-day feeding period abrogated oral tolerance (Fig. 1a; Supplemental Fig. 1b), which is consistent with a previous study [21]. Using 16S rRNA sequencing, we confirmed that the gut microbiota was markedly depleted following 3 days of antibiotic treatment and that many major groups of bacteria were restored after the cessation of antibiotics (Supplemental Fig. 1c, d).

We next examined the effect of antibiotics on immune cells in the SILP of naive mice by flow cytometry. For this, WT mice were treated with antibiotics for 5 days and flow cytometric analysis performed the day after (Fig. 1b, left panel). CX3CR1^+^ mononuclear phagocytes (MNPs) play an important role in the induction of tolerance by transferring orally administered antigens to tolerogenic CD103^+^ dendritic cells (DCs) [22, 23] and by secreting IL-10 that maintains Treg activation/survival and decreases Th17 cells in the gut [24–27]. We found that antibiotics increased both CD11c^+^CD11b^+^CX3CR1^+^ MNPs and CD11c^+^CD11b^−^CD103^+^ DCs in the SILP (Fig. 1c), suggesting that these cell frequencies may be elevated due to a compensatory effect in antibiotic-treated mice. In the colon, which is not a site for oral tolerance induction, we found lower CX3CR1^+^ MNPs and no changes in CD103^+^ DCs following microbiota depletion (Fig. 1c). We then investigated γδ T cells, which are critical for the induction of oral tolerance [28–31] and which can be shaped by the microbiota [1, 9, 32]. We found that antibiotics decreased γδ T cells in both lamina propria and intraepithelial lymphocyte (IEL) compartments, particularly in the small intestine (Fig. 1d). This suggests that the microbiota is needed to maintain γδ T cells in the gut and that this requirement may account for the loss in tolerance induction following antibiotic treatment.

To identify potential immunologic pathways related to the loss of tolerance in antibiotic-treated mice, we performed gene expression analysis of the whole tissue duodenum and ileum using the NCounter Mouse Immunology panel (Fig. 1b, right panel, Fig. 1e–g; Supplemental Table 1). We found that antibiotics affected similar pathways in both sites, with 208 genes altered in the duodenum (13 down, 195 up), 158 genes altered in the ileum (18 down, 140 up), and greater than 100 genes shared (Fig. 1d). Using Ingenuity Pathway Analysis, we found that antibiotics increased genes related to DC maturation, NFAT immune regulation, and NF-KB, Th17, STAT3, IL6, and CD40 signaling pathways (Fig. 1e). Antibiotics decreased PPARa/RXRa activation, PTEN signaling, and antioxidant action of vitamin C-related pathways (Fig. 1e). In both duodenum and ileum, we found elevated gene expression involved in immune activation and inflammatory immune responses, including *Cd86*, *Il1a*, *Il6ra*, and *Icosl* (Fig. 1f). Consistent with our flow cytometry results (Fig. 1b), we found that antibiotics increased *Cx3cr1* expression in the duodenum and ileum (Fig. 1g). We found that antibiotics decreased the expression of *Cd55* in the duodenum and ileum (Fig. 1g); CD55 has been shown to be essential for the tolerogenic function of CD103^+^ DCs [33]. Furthermore, we found that antibiotics decreased the expression of *Ccl25* and butyrophilin-like 1 (*Btnl1*), factors that recruit γδ T cells to the small intestine and shape the intestinal γδ T cell compartment, respectively [34, 35]. This may account for the reduced numbers of γδ T cells we observed in the small intestine (Fig. 1g). Taken together, these results suggest that the microbiota promotes oral tolerance by restraining inflammatory responses in the gut and by maintaining populations of γδ T cells.

### Lack of γδ T cells leads to intestinal immune dysfunction and impairs oral tolerance

**Fig. 2:**
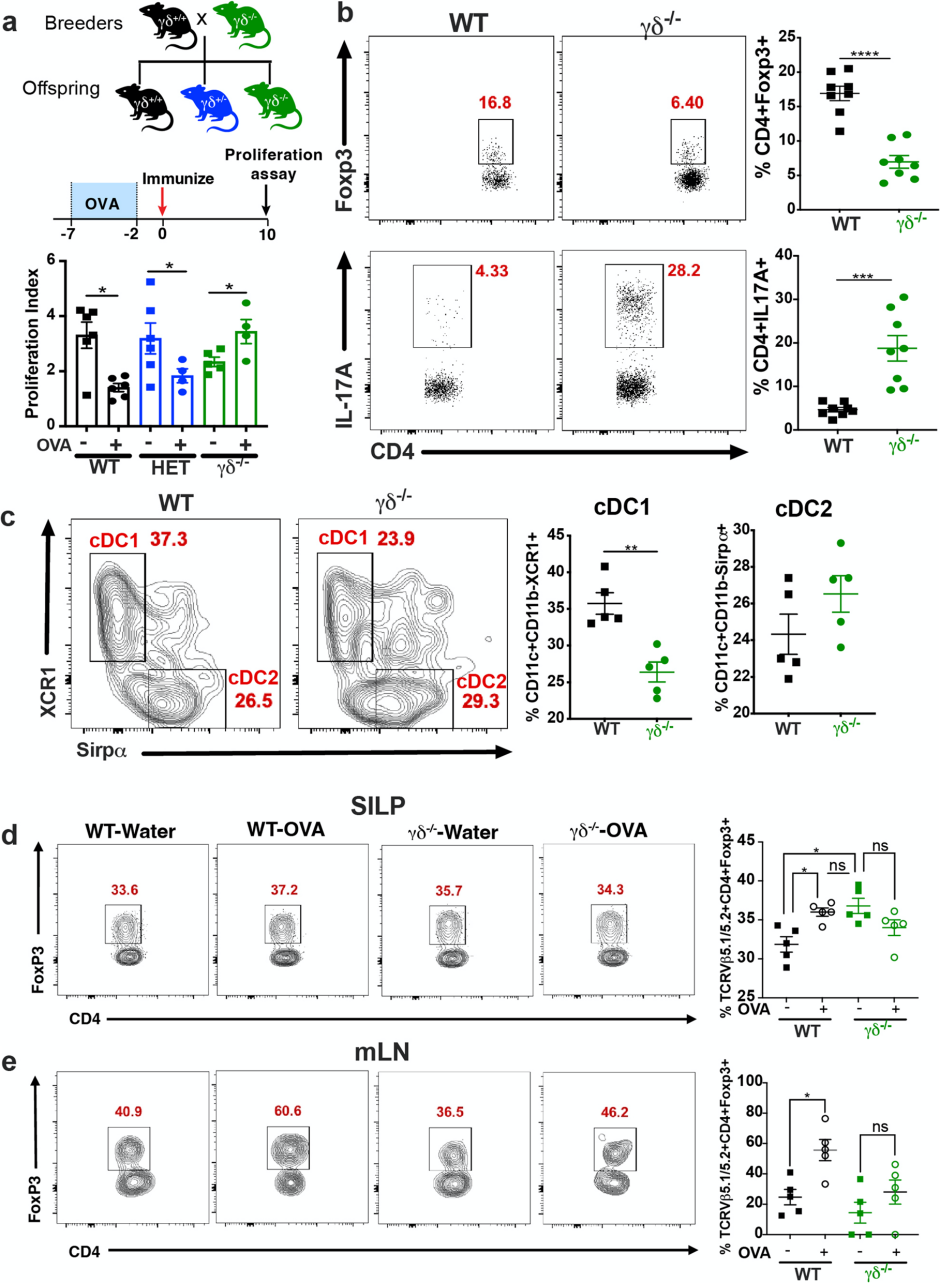
Intestinal immune dysregulation and impaired oral tolerance in γδ^−/−^ mice. **a** Scheme for γδ^+/−^ × γδ^+/−^ breeding strategy and oral tolerance induction. Mice were fed OVA in the drinking water for 5 days. OVA continuous feeding was stopped and 2 days later mice were immunized with OVA/CFA. Responsiveness to OVA was measured by splenocyte proliferation upon 100 µg/mL of OVA stimulation. Data are mean + SEM; *n*=5 mice/group; one-way ANOVA. **b, c** FACS plots and bar graphs showing frequencies of live CD3^+^CD4^+^Foxp3^+^ and CD3^+^CD4^+^IL-17A^+^ in the small intestine lamina propria (SILP; **c**), and migratory cDC1s (MHC-II^high^CD11c^+^CD11b^−^CD103^+^XCR1^+^Sirpα^−^) and migratory cDC2s (MHC-II^high^CD11c^+^CD11b^+^CD103^+^XCR1^−^Sirpα^+^) from the mesenteric lymph node (mLN; **c**) of γδ^−/−^ and WT mice before OVA feeding. **d, e** FACS plots and bar graphs showing frequencies of live OVA-specific Treg cells (Vβ5.1/5.2^+^CD4^+^Foxp3^+^) in the mLN (**d**) and in the SILP (**e**) of γδ^−/−^ and WT mice 2 days after 5 days of OVA continuous feeding in the drinking water. Data are mean + SEM; *n*=5–8 mice/group; one-way ANOVA. *p < 0.05, ** *p* < 0.01, *** *p* < 0.001, **** *p* < 0.0001. Results are representative of at least two independent experiments

γδ^−/−^ mice have been shown to develop more severe wasting disease in several colitis models [5, 10, 11]. Moreover, gd T cells have been shown to play an important role in mucosal tolerance induction [28–31]. To investigate the role of γδ T cells in intestinal homeostasis, we bred heterozygous γδ^+/−^ mice to generate γδ^+/+^ (WT),  gdγδ^+/−^ (HET) and γδ^−/−^ mice and measured oral tolerance induction against OVA. This breeding strategy is important to rule out any cage and animal facility effect that may occur when offspring are generated from homozygous breeders. WT, HET, and γδ^−/−^ mice were fed OVA in the drinking water for 5 consecutive days; immunized with OVA emulsified in complete Freund’s adjuvant (CFA) 2 days later; and spleens removed 10 days after immunization for proliferation assay. As shown in Fig. 2a, while WT and HET mice developed tolerance against OVA, as measured by reduced splenocyte proliferation when stimulated with OVA in vitro, γδ^−/−^ mice failed to develop oral tolerance. This suggests that the tolerogenic functions of the small intestine (the primary site for oral tolerance induction) of γδ^−/−^ mice is impaired and that the lack of γδ T cells rather than an unspecific cage effect was responsible for oral tolerance impairment. Since oral tolerance does not develop in an inflamed intestinal milieu [36], we next investigated whether the small intestine of γδ^−/−^ mice was inflamed. Of note, we only used WT and γδ^−/−^ mice since no difference in oral tolerance was observed between HET vs. WT mice (Fig. 2a). We found that γδ^−/−^ mice had decreased frequencies of Foxp3^+^ regulatory T (Treg) cells and increased frequencies of IL-17-producing CD4^+^ T (Th17) cells (Fig. 2b). Of note, γδ^−/−^ mice do not show inflammation in the gut at steady state as observed by histopathological analysis [5]. Thus, this shift in Treg/Th17 balance indicates an immune dysregulation, rather than overt inflammation in the intestinal mucosal. Consistent with this, we also found reduced frequencies of IL-10-expressing CD4+ T cells (Supplemental Fig. 2a), but no difference in CD4^+^IFN-γ^+^ (Th1) (Supplemental Fig. 2b), CD8^+^IFN-γ^+^ (Supplemental Fig. 2c), and CD8^+^IL17A^+^ (Supplemental Fig. 2d) cells between WT and γδ^−/−^ mice.

An imbalance between Th17 and Treg cells may be associated with an impaired migration of tolerogenic DCs to the mesenteric lymph node (mLN) and the subsequent induction of Treg cells [37]. It has been proposed that two subtypes of migratory tolerogenic DCs induce Treg cell differentiation in the mLN: CD11c^+^CD11b^−^CD103^+^XCR1^+^ DCs, also known as cDC1s, and CD11c^+^CD11b^+^ CD103^+^Sirpα^+^ DCs, also known as cDC2s [37]. These DCs take up antigen in the SILP and migrate to the mLN to induce Treg cell differentiation [37]. To investigate whether reduced frequencies of Treg cells in the SILP of γδ^−/−^ mice was associated with reduced migration of cDC1 and/or cDC2 to the mLN, we isolated migratory (CD11c^+^MHCII^high^) cDC1s and cDC2s from the mLN and performed flow cytometric analysis. We found a significant reduction in cDC1s, but not cDC2s, in ^−/−^ mice as compared to WT mice (Fig. 2c), indicating that impaired Treg cell differentiation in the mLN may play a role in the imbalance of Th17/Treg frequencies in the gut of γδ^−/−^ mice. To test this hypothesis, γδ^−/−^ and WT mice were fed OVA in the drinking water for 5 days and euthanized 2 days later for mLN flow cytometric analysis of OVA-specific Treg cells by using anti-Vβ5.1/5.2 monoclonal antibody, which stains T cell-expressing OVA-specific TCRβ chain. We found that while WT mice fed OVA had increased frequencies of OVA-specific Treg cells (Vβ5.1/5.2^+^CD4^+^Foxp3^+^) compared to control WT group, control γδ^−/−^ mice (fed water) had already increased frequencies of Tregs that did not change following oral administration of OVA (Fig. 2d). This suggests that Treg cell differentiation is not impaired in the mLN of γδ^−/−^ mice. We then investigated whether OVA-specific Treg cells were decreased in the SILP of γδ^−/−^ mice, which would indicate that the activation and/or survival of Treg cells is impaired in these mice. Consistent with this, we found that while OVA-specific Treg cells were increased in the LP of WT mice fed OVA, these cells were decreased in γδ^−/−^ mice fed water and did not increase following OVA feeding (Fig. 2e). These findings suggest that activation/survival of Treg cells in the SILP rather than Treg cell differentiation in the mLN is responsible for the impaired oral tolerance induction in γδ^−/−^ mice.

Taken together, these data suggest that γδ T cells are necessary to shape intestinal immune tolerogenic populations to prevent immune dysregulation in the gut.

### Altered microbiota in γδ^−/−^ mice induces intestinal immune dysregulation

**Fig. 3:**
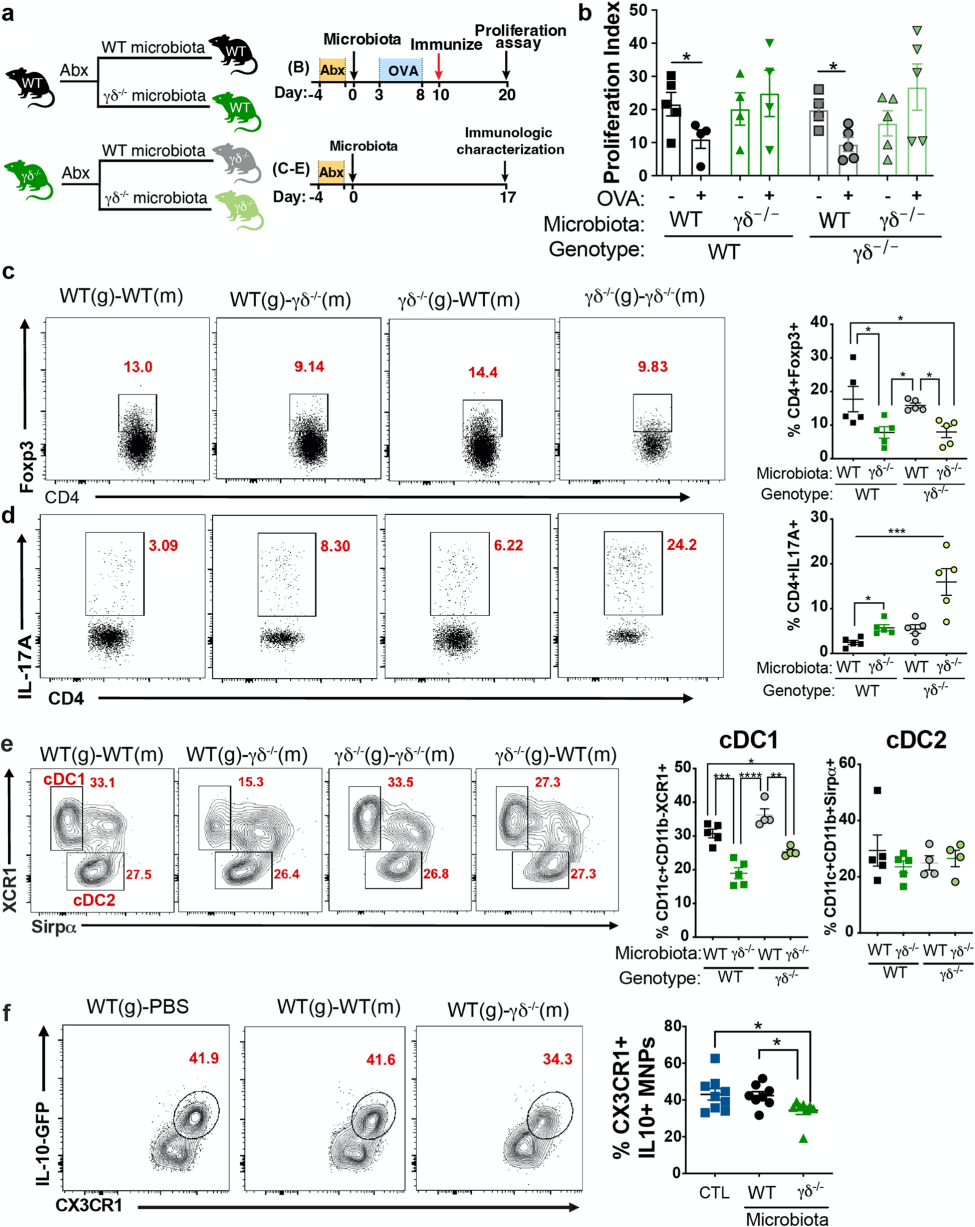
Defective oral tolerance in γδ^−/−^ mice is mediated by the gut microbiota. **a** Microbiota was depleted with a combination of 4 antibiotics (ABX) in the drinking water for 3 days and 1 day later, microbiota from WT and γδ^−/−^ mice were swapped. To assess oral tolerance, 3 days later, half of each group were fed OVA in the drinking water for 5 days. OVA continuous feeding was stopped and 2 days later mice were immunized with OVA/CFA. In an independent cohort, the immune responses were characterized 17 days post colonization. **b** Oral tolerance to OVA was measured by splenocyte proliferation upon 100 µg/mL of OVA stimulation. Data are mean + SEM; *n*=5 mice/group; one-way ANOVA. **c–e** FACS plots and bar graphs showing frequencies of live CD3^+^CD4^+^Foxp3^+^ (**c**) CD3^+^CD4^+^IL-17A^+^ (**d**) in the small intestine lamina propria (SILP), and migratory cDC1s (MHC-II^high^CD11c^+^CD11b^−^CD103^+^XCR1^+^Sirpα^−^) and cDC2s (MHC-II^high^CD11c^+^CD11b^+^CD103^+^XCR1^−^Sirpα^+^) (**e**) from the mesenteric lymph node (mLN) of mice colonized with WT or γδ^−/−^ microbiota. (**f**) FACS plots and bar graphs showing frequencies of live CD11b^+^CD103^−^CX3CR1^+^IL-10^+^ mononuclear phagocytes (MNPs) in the SILP of IL-10-GFP reporter mice treated with broad-spectrum antibiotics or left untreated (for mice treated with PBS) for 3 days and colonized 2 days later with WT or γδ^−/−^ microbiota or treated with PBS. Data are mean ± SEM; *n*=5–8 mice/group; one-way ANOVA. * *p* < 0.05, ** *p* < 0.01, *** *p* < 0.001, **** *p* < 0.0001. Results are representative of at least two independent experiments

Intestinal γδ T cells have the important role of preventing microbial invasion to the gut, which maintains intestinal homeostasis [9]. We hypothesized that the absence of γδ T cells would lead to an altered microbial community, which in turn would induce gut immune dysregulation and impair mucosal tolerance. To address this, we first treated mice with antibiotics in their drinking water, as described above, colonized mice with cecal microbiota from WT or γδ^−/−^ mice and performed oral tolerance experiments (Fig. 3a). We found that colonization of WT mice with γδ^−/−^ microbiota impaired oral tolerance induction whereas colonization of γδ^−/−^ mice with WT microbiota restored oral tolerance (Fig. 3b). Moreover, flow cytometric analysis of SILP cells 3 weeks after microbial colonization and before oral tolerance induction showed that WT mice colonized with γδ^−/−^ microbiota had decreased levels of Treg cells and tolerogenic cDC1s, and increased levels of Th17 cells in the LP (Fig. 3c–e). In contrast, colonization of γδ^−/−^ mice with WT microbiota restored Treg, Th17, and cDC1 to normal levels (Fig. 3c–e).

As mentioned above, Treg cell activation/survival in the SILP rather than Treg cell differentiation in the mLN is likely involved in the impaired mucosal tolerance observed in γδ^−/−^ mice. An imbalance between Th17 and Treg cells may be associated with decreased IL-10 release from CX3CR1^+^ mononuclear phagocytes (MNPs), which are major producers of IL-10 in the gut [26]. Moreover, decreased IL-10 production by CX3CR1^+^ MNPs has been associated with intestinal inflammation and impaired oral tolerance induction due to defective Treg activation and survival [24–27]. We then hypothesized that γδ^−/−^ dysbiotic microbiota would lead to a decrease in IL-10 production by CX3CR1^+^ MNPs, which in turn would affect Treg/Th17 balance in the SILP. To test this hypothesis, we treated IL-10-GFP mice with antibiotics for 3 days and then colonized mice with WT or γδ^−/−^ microbiota. Of note, we used IL-10-GFP mice because IL-10 staining with anti-IL-10 monoclonal antibody did not lead to consistent results in our flow cytometry analysis (not shown). We found that IL-10 production by CX3CR1^+^ MNPs was reduced in mice transferred with γδ^−/−^ microbiota (Fig. 3f), suggesting that microbes from γδ^−/−^ mice negatively affect IL-10 producing CX3CR1^+^ MNPs, which leads to an immune dysregulation in the gut.

We then investigated changes in the microbiome by 16S rRNA sequencing of the V4 region at 17 days post colonization in the duodenum, ileum, cecum, and colon (Supplemental Fig. 3a). By performing principal coordinate analysis (PCoA), we found that microbes clustered by microbiota inoculum source, host genotype, and anatomical location (Supplemental Fig. 3b). By the ADONIS test, microbiota inoculum source accounted for 23.1% of the variation (*p*< 0.001), genotype accounted for 8.50% of the variation (*p*<0.001), and anatomical location accounted for 24.7% of the variation (*p*<0.001) (Supplemental Fig. 3c). Using linear discriminant analysis effect size (LEfSe), we asked which microbes were altered by microbiota treatment (class) within each recipient genotype (subclass). We found that *Ruminococcus gnavus*, *Bilophila*, *Bifidobacterium*, and *Akkermansia* were decreased in mice colonized with γδ^−/−^ microbiota whereas *Desulfovibrio*, *Helicobacter hepaticus*, and *Candidatus Arthromitus* were increased (Supplemental Fig. 3d, e). Taken together, these data indicate that impaired intestinal tolerogenic functions of the γδ^−/−^ mice are a consequence of a dysbiotic microbial community that develops in the absence of γδ T cells and induces gut immune dysregulation.

**Fig. 4:**
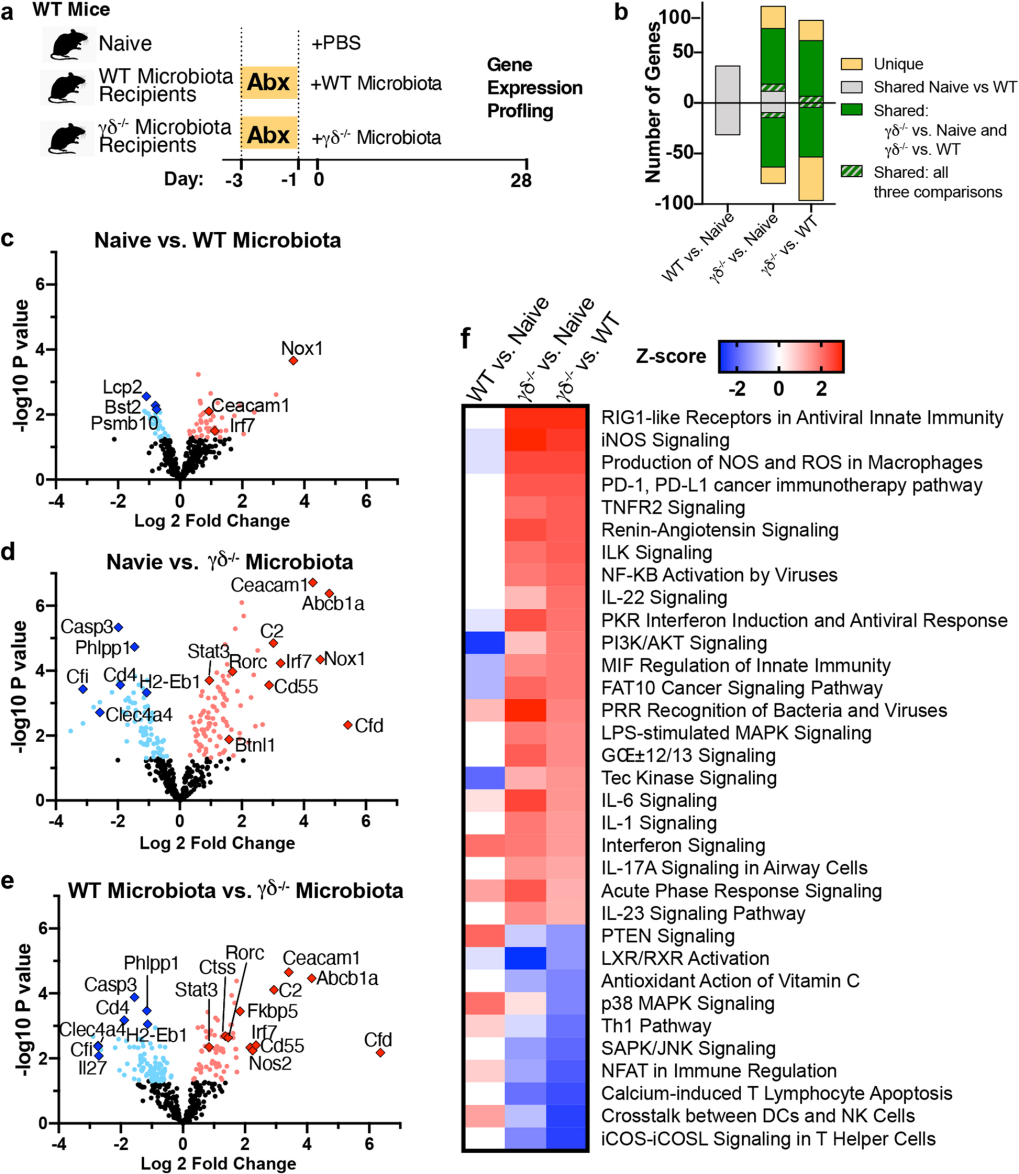
γδ^−/−^ microbiota induces an inflammatory intestinal milieu. **a** Microbiota was depleted in WT recipient mice with a combination of 4 antibiotics (ABX) in the drinking water for 3 days and 1 day later they were colonized or not (CTL) with either WT or γδ^−/−^ microbiota. Mice were sacrificed 3 weeks later, and the duodenum collected for Nanostring analysis using the NCounter Mouse Immunology Panel; *n*=5 mice/group. **b** Number of genes shared by and unique to naive vs. WT microbiota-colonized mice and by naive and WT microbiota-colonized mice with mice colonized with γδ^−/−^ microbiota. **c–e** Volcano plots of genes significantly altered in the duodenum of naive vs. WT microbiota-colonized mice (**c**), naive vs. γδ^−/−^ microbiota-colonized mice (**d**), and WT microbiota vs. γδ^−/−^ microbiota-colonized mice (**e**). NCounter advanced analysis module, *p* < 0.05. **f** Canonical pathways altered by antibiotic treatment determined by Ingenuity Pathway. Results are representative of at least two independent experiments

To further investigate the mechanisms by which γδ^−/−^ microbiota induces immune dysregulation, we profiled 547 immune-related transcripts in the SILP of antibiotic-treated WT mice colonized with γδ^−/−^ or WT microbiota (Fig. 4a; Supplementary Table 2). To establish a baseline, we first measured gene expression in antibiotic-treated WT mice colonized with WT microbiota vs. naïve mice and found relatively few genes altered (Fig. 4b, c), indicating that antibiotic treatment and microbial transfer only slightly affected transcriptional signatures. This allowed us to identify genes independent of antibiotic treatment and γδ^−/−^ microbiota transfer, including *Ceacam1* and *Lcp2*, and to separate these from genes associated with γδ^−/−^ microbiota transfer. We then compared gene changes in (1) antibiotic-treated WT mice colonized with γδ^−/−^ microbiota vs. naïve mice (Fig. 4d) and (2) antibiotic-treated WT mice colonized with γδ^−/−^ microbiota vs. antibiotic-treated WT mice colonized with WT microbiota (Fig. 4e). We found over 150 genes altered with a majority shared in both comparisons against γδ^−/−^ microbiota recipients (Fig. 4b). γδ^−/−^ microbiota increased *Rorc* and *Stat3* (Fig. 4d, e), which are transcription factors that favor Th17 cell differentiation and inhibit Treg cell induction [38, 39]. We then performed Ingenuity Pathway Analysis and as shown in Fig. 4f, mice colonized with γδ^−/−^ microbiota upregulated pro-inflammatory pathways including LPS-stimulated MAPK signaling, pattern recognition receptors, recognition of bacteria and viruses, production of reactive oxidative species in macrophages, and increased IL-1β, IL-6, IL-23, and TNF-α signaling. Of note, IL-6, IL-1β, and IL-23 are known to induce Th17 and inhibit Treg cell differentiation [38, 39]. On the other hand, iCOS-iCOSL signaling in helper T cells, NFAT in immune regulation, and the Th1 pathway were decreased in mice receiving γδ^−/−^ microbiota. Taken together, these results indicate that microbiota from γδ^−/−^ mice induces an inflammatory milieu in the small intestine most likely triggered by exposure to pro-inflammatory microbial products. This is consistent with our finding that γδ^−/−^ microbiota promotes an imbalance between Treg and Th17 cells in the small intestine. Thus, these findings demonstrate that γδ T cells select for an intestinal microbiota that is needed for mucosal tolerance.

### *Ruminococcus gnavus* restores oral tolerance in γδ^−/−^ mice

**Fig. 5:**
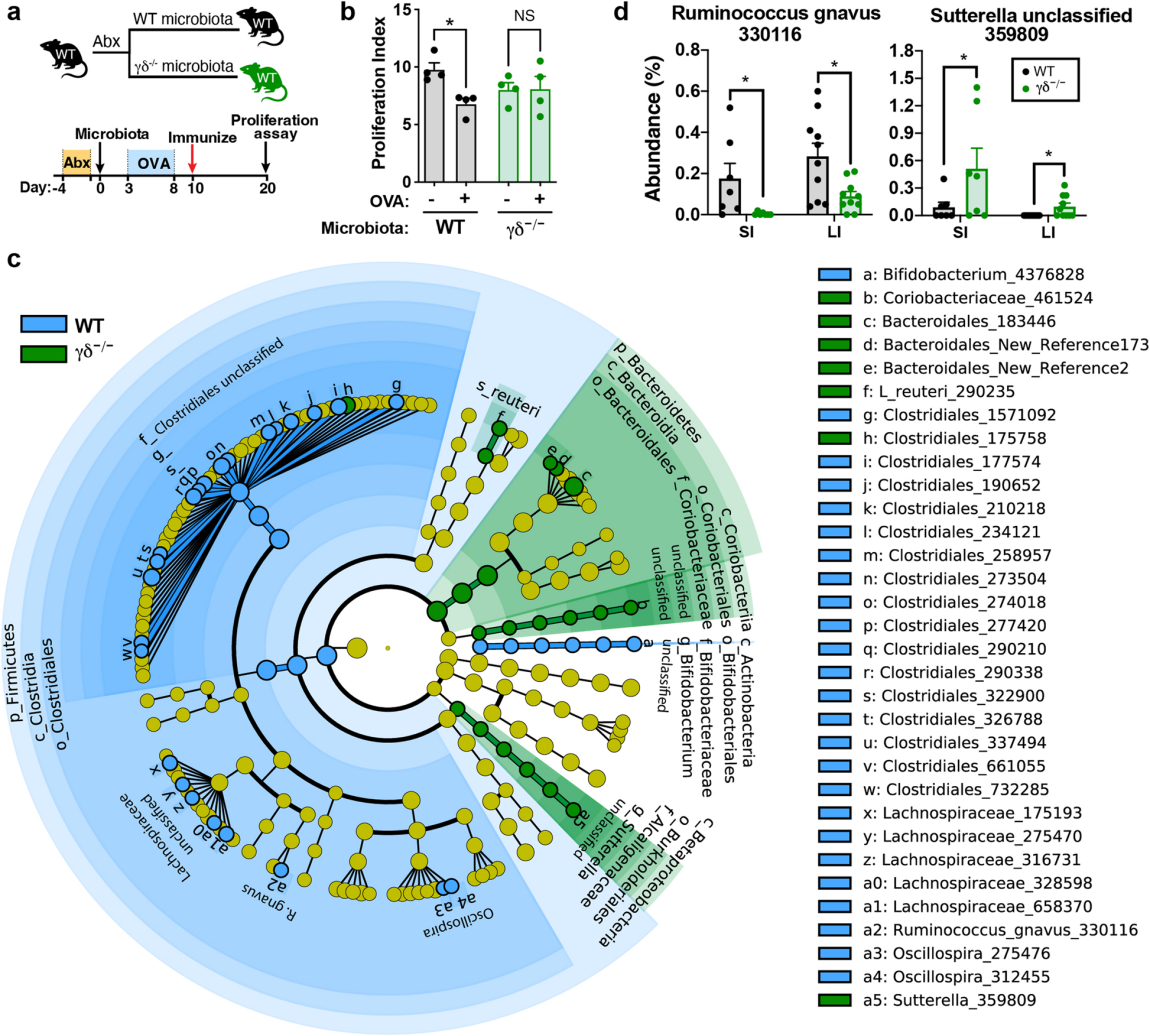
γδ^−/−^ microbiota is deficient in *R. gnavus* and enriched in *P. excrementihominis*. **a** Scheme for antibiotic and microbiota colonization. WT mice were treated with broad-spectrum antibiotics for 3 days, transferred with either WT or γδ^−/−^ microbiota 2 days later and continuously fed OVA (or only water as control) starting 3 days following microbiota transfer and ending 5 days later. Mice were then immunized with OVA/CFA 2 days after the last dose of OVA. **b** Responsiveness to OVA was measured by splenocyte proliferation upon 100 µg/mL of OVA stimulation. Data are mean ± SEM; *n*=4 mice/group; one-way ANOVA. **c** Cladogram representing all taxa detected at > 0.1%, shown at the Kingdom phylogenetic level through the genus level LEfSe *p* < 0.05. Yellow circles depict taxa present, but not enriched. Blue and green circles represent microbes that are enriched in WT and γδ^−/−^ microbiota, respectively. The size of the circle corresponds to the population of each taxon. Kruskal-Wallis one-way analysis was used for microbiome analysis

We then asked whether we could identify specific components of the microbiome that were consistently associated with defective mucosal tolerance and whether administering an individual bacterium could restore tolerance. We transferred γδ^−/−^ and WT microbiota to an independent cohort of antibiotic-treated WT mice and again found that γδ^−/−^ microbiota impaired oral tolerance **(**Fig. 5a, b). We sequenced the V4 region of the 16S rRNA gene from intestinal samples from this second cohort of mice and measured differences in the small and large intestines by linear discriminant analysis effect size (LEfSe) [18]. We found several bacteria that were either decreased or increased in γδ^−/−^ microbiota (Fig. 5c, d; Supplemental Fig. 4a). Consistent with the microbiota transfer experiment findings described above, we found that *Ruminococcus gnavus* was decreased in γδ^−/−^ mice (Fig. 5d). Moreover, we found that an unclassified *Sutterella* species, which had a high homology to *Parasutterella excrementihominis*, was increased in γδ^−/−^ mice (Fig. 5d). Furthermore, *R. gnavus* is an attractive candidate for a bacterium that might enhance oral tolerance as it is a mucus-degrading bacterium that facilitates bacterial adherence [40–42] and it is known that bacterial adherence to the gut epithelium is critical for oral tolerance [26]. *P. excrementihominis* is a member of the *Sutterellaceae* family and has been associated with inflammatory bowel diseases (IBD) [43].

We then examined microbial function from γδ^−/−^ mice by generating a predicted metagenome from the microbial 16S rRNA sequences using phylogenetic investigation of communities by reconstruction of unobserved states (PICRUSt) [19]. A large number of microbial Kyoto Encyclopedia Genes and Genomes (KEGG) pathways was altered in γδ^−/−^-microbiota recipients compared to WT microbiota recipients. As shown in Supplemental Fig. 4b-d, impaired oral tolerance was associated with a decrease in pathways related to bacterial motility and transport, suggesting a lack of microbe-host interactions that promote tolerogenic immunologic function. Conversely, we found increased pathways related to lipopolysaccharide (LPS) biosynthesis, a key component of Gram-negative cell walls that can stimulate inflammatory responses.

**Fig. 6:**
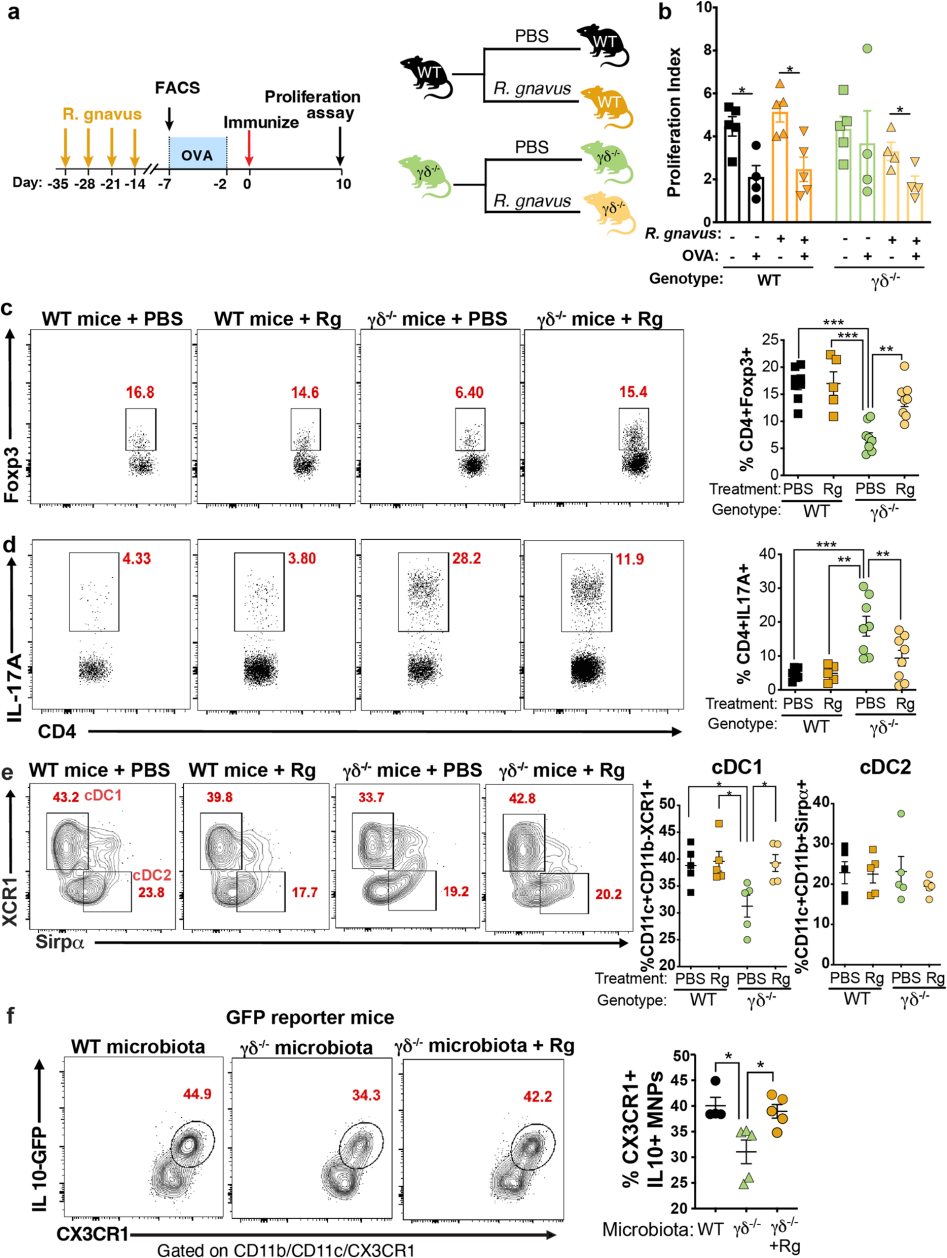
*R. gnavus* restores oral tolerance in γδ^−/−^ mice. **a** WT and γδ^−/−^ mice were gavaged with *R. gnavus* once a week for 4 weeks. One week later, half of each group were fed OVA in the drinking water for 5 days. OVA continuous feeding was stopped and 2 days later mice were immunized with OVA/CFA. **b** Responsiveness to OVA was measured by splenocyte proliferation upon 100 µg/mL of OVA stimulation. Data are mean ± SEM; *n*=4–5 mice/group; one-way ANOVA. **c–e** FACS plots and bar graphs showing frequencies of live CD3^+^CD4^+^Foxp3^+^ (**c**) and CD3^+^CD4^+^IL-17A^+^ (**d**) from small intestine lamina propria (SILP), and migratory cDC1s (MHC-II^high^CD11c^+^CD11b^−^CD103^+^XCR1^+^Sirpα^−^) and cDC2s (MHC-II^high^CD11c^+^CD11b^+^CD103^+^XCR1^−^Sirpα^+^) (**e**) from the mesenteric lymph node (mLN) of mice colonized or not (PBS) with *R. gnavus* before OVA/CFA immunization. **e** FACS plots and bar graphs showing frequencies of live CD11b^+^CD103^−^CX3CR1^+^IL-10^+^ mononuclear phagocytes (MNPs) in the SILP of IL-10-GFP reporter mice treated with broad-spectrum antibiotics for 3 days and colonized 2 days later with WT microbiota, γδ^−/−^ microbiota or γδ^−/−^ microbiota + *R. gnavus* (Rg). Data are mean ± SEM; *n*=5–10 mice/group; one-way ANOVA. * *p* < 0.05, ** *p* < 0.01, *** *p* < 0.001. Results are representative of at least two independent experiments

To test the hypothesis that *R. gnavus* would restore oral tolerance and *P. excrementihominis* would impair oral tolerance, WT and γδ^−/−^ mice were colonized with either *R. gnavus* or *P. excrementihominis* once weekly for 4 weeks, and oral tolerance was induced by feeding OVA (Fig. 6a). We found that *R. gnavus* colonization rescued oral tolerance in γδ^−/−^ mice but did not affect oral tolerance in WT mice (Fig. 6b). On the other hand, when we colonized WT mice with *P. excrementihominis* (Supplemental Fig. 4e), we did not find that oral tolerance was impaired (Supplemental Fig. 4f) even though we increased *P. excrementihominis* in WT mice to levels seen in γδ^−/−^ mice (Supplemental Fig. 4g). Thus, decreased *R. gnavus* rather than increased *P. excrementihominis* in γδ^−/−^ mice is associated with impaired oral tolerance in these mice.

We then investigated immune effects associated with restoration of oral tolerance by *R. gnavus* in γδ^−/−^ mice. As shown in Fig. 6c, d, there are decreased Tregs and increased Th17 cells in the SILP of γδ^−/−^ mice and we found that *R. gnavus* colonization normalized these frequencies. Consistent with this, we found that *R. gnavus* colonization also restored cDC1 migratory properties to the mLN (Fig. 6e). To investigate whether colonizing γδ^−/−^ mice with *R. gnavus* restored IL-10 production by CX3CR1^+^ MNPs, we treated IL-10-GFP mice with ABX for 3 days and then colonized mice with WT, γδ^−/−^ or γδ^−/−^ microbiota + *R. gnavus*. Consistent with our findings shown in Fig. 3f, IL-10 production by CX3CR1^+^ MNPs was reduced in mice transferred with γδ^−/−^ microbiota, but IL-10 levels were restored when IL-10-GFP mice received γδ^−/−^ microbiota + *R. gnavus* (Fig. 6f). Thus, *R. gnavus* rescues mucosal tolerance in γδ^−/−^ mice by restoring intestinal homeostasis.

### *Ruminococcus gnavus* regulates intestinal mucus layer

It has been demonstrated that the intestinal epithelial barrier is critical for oral tolerance and that microbial adherence plays an important role in gut homeostasis [26]. Microbial adherence promotes IL-10 production by CX3CR1^+^ MNPs, which plays an important role in Treg/Th17 balance [24–27]. A critical factor that regulates microbial adherence to the gut epithelium is the mucus. A thick mucus layer affects the interaction between microbes and intestinal epithelial cells [44]. Because *R. gnavus* is a mucus degrader and because it is reduced in γδ^−/−^ microbiota, we hypothesized that γδ^−/−^ mice would develop a thicker mucus layer and that this thicker layer would impair mucosal tolerance. To investigate this hypothesis, we performed histological analysis of small intestine of γδ^−/−^ mice using periodic acid-Schiff (PAS), a mucus staining compound. We found that γδ^−/−^ mice have a thicker mucus layer as compared to WT mice (Supplemental Fig. 5a, b). Moreover, colonization of γδ^−/−^ mice with *R. gnavus* normalized the mucus layer thickness (Supplemental Fig. 5a, b). These results were further confirmed by staining γδ^−/−^ mouse small intestine with wheat germ agglutinin (WGA), a carbohydrate-binding lectin with high affinity for sialic acid and N-acetylglucosamine, which are mucus constituents (Supplemental Fig. 5c).

Taken together, these findings suggest that γδ^−/−^ mice, due to the lack of *R. gnavus*, develop an increased intestinal mucus layer that may affect a physiologic microbial adherence to the epithelium, which leads to reduced IL-10 production by CX3CR1^+^ MNPs [26].

### γδ T cells respond to altered microbiota by secreting AMPs and the miRNA let-7f

One mechanism by which intestinal cells shape the gut microbiota is through secretion of antimicrobial peptides (AMPs) [6–8]. AMPs are a class of small peptides with a wide range of inhibitory effects against bacteria, parasites, and viruses [45]. Several AMPs have been described, including members of the regenerating islet-derived (Reg) protein family of C-type lectins such as RegIIIα, RegIIIβ, and RegIIIγ as well as defensins. It has been shown that intestinal γδ T cells induce gut epithelial cells to secrete AMPs [9]. To investigate the expression levels of RegIII mRNAs in WT vs. γδ^−/−^ mice at steady state, we sorted αβ T cells from SILP and SI-IEL compartments as well as epithelial cells (EpCAM^+^) from the small intestine, and measured RegIII mRNAs by RT-qPCR. We found that while no difference was observed in *Reg3a*, *Reg3b,* and *Reg3g* mRNA expression levels in αβ T cells isolated from SILP or SI-IEL between WT and γδ^−/−^ mice, epithelial cells from γδ^−/−^ mice upregulated both *Reg3b* and *Reg3g* mRNAs (Supplemental Fig. 6a-c). These findings suggest that γδ T cells may play an important role in secreting AMPs into the gut lumen because, in the absence of γδ T cells, epithelial cells compensate for RegIIIβ and RegIIIγ secretion.

To investigate whether γδ T cells secreted AMPs, we treated 4-week-old WT mice with antibiotics in the drinking water for 3 days and then colonized mice with cecal microbiota from WT or γδ^−/−^ mice. Three weeks later, we performed RT-qPCR for Reg3a, Reg3b, and Reg3g from αβ and γδ T cells sorted from intestinal LP and IEL compartments. We found that transfer of γδ^−/−^ microbiota to WT mice increased the expression of *Reg3b* and *Reg3g* mRNAs in γδ, but not αβ T cells from the SI-IEL compartment (Supplemental Fig. 6d). No difference was observed in *Reg3a*, *Reg3b*, and *Reg3g* mRNA levels in the SILP (not shown).

**Fig. 7:**
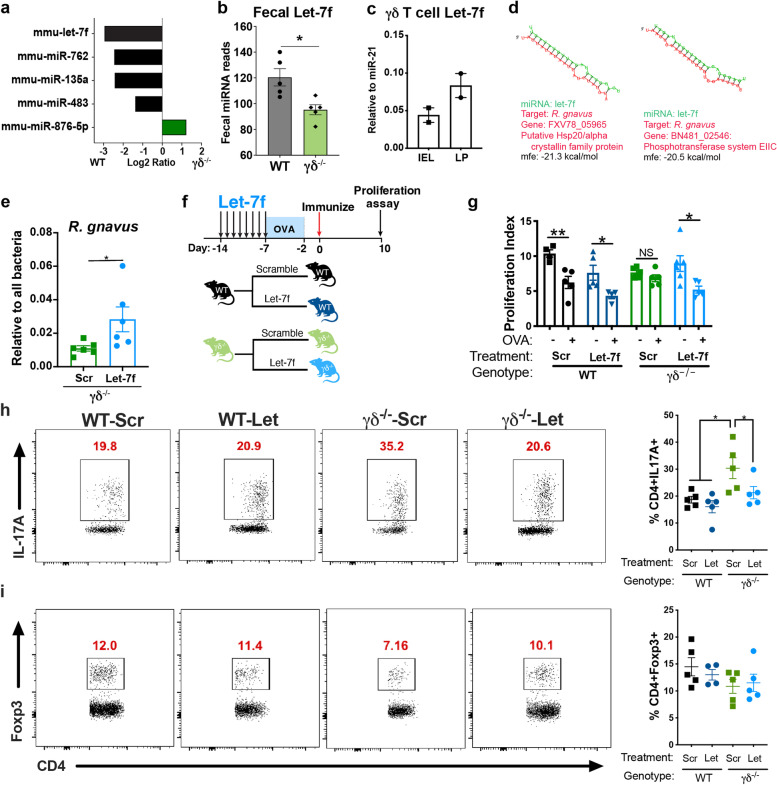
Intestinal γδ T cells secrete miRNAs to promote *Ruminococcus gnavus* growth and restore oral tolerance. **a, b** Nanostring miRNA analysis of statistically different up and downregulated fecal miRNAs. γδ^−/−^/WT Log2 ratio with *p* < 0.05 is shown (**a**) and total reads of let-7f in the feces from WT and γδ^−/−^ mice (**b**); *n*=5 mice/group; Student’s *t* test. **c** IEL and LP γδ T cells were sorted and let-7f expression measured by RT-qPCR. Relative expression to miR-21 (the most abundant fecal miRNA) is shown. Data are mean ± SEM; *n*=4 mice pooled into 2 samples. **d** Bacterial genes of *R. gnavus* that are predicted to be targeted by let-7f, indicated by sequence blast and predicted for secondary structure property (minimum free energy, mfe) by RNAhybrid. **e** Let-7f or its scrambled sequence (Scr) were orally given to WT and γδ^−/−^ mice for 7 consecutive days at a dose of 1000 pmol/mouse/day. On the last day of miRNA administration, fecal samples from WT and γδ^−/−^ mice were collected and RT-qPCR performed to detect *R. gnavus* levels. Data are mean ± SEM; *n*=6 mice/group; Student’s *t* test. **f** Let-7f or its scrambled sequence (Scr) were orally given to WT and γδ^−/−^ mice for 7 consecutive days at a dose of 1000 pmol/mouse/day. On the last day of miRNA administration, half of each group were fed OVA in the drinking water for 5 days. OVA continuous feeding was stopped and 2 days later mice were immunized with OVA/CFA. **g** Responsiveness to OVA was measured by splenocyte proliferation upon 100 µg/mL of OVA stimulation 10 days after OVA/CFA immunization. Data are mean ± SEM; *n*=5–6 mice/group; one-way ANOVA. **h, i** FACS plots and bar graphs showing frequencies of live CD3^+^CD4^+^Foxp3^+^ (**h**) and CD3^+^CD4^+^IL-17A^+^ (**i**) from small intestine lamina propria (SILP) of mice orally treated with let-7f or its scrambled sequence one day after the last gavage and before OVA/CFA immunization. Data are mean ± SEM; *n*=5 mice/group; one-way ANOVA. NS=non-significant, * *p* < 0.05, ** *p* < 0.01, *** *p* < 0.001. Results are representative of at least two independent experiments

We have recently demonstrated that fecal miRNAs secreted by intestinal immune cells including epithelial and dendritic cells play an important role in shaping the gut microbiota, which contributes to intestinal homeostasis [13, 46]. To investigate whether intestinal γδ T cells also secrete fecal miRNAs that could contribute to microbiota regulation, we performed a miRNA Nanostring in the feces of γδ^−/−^ mice and found that four miRNAs (let-7f, miR-762, miR-135a, and miR-483) were downregulated and one miRNA (miR-876-5p) was upregulated in γδ^−/−^ feces vs. WT animals (Fig. 7a, b; Supplemental Table 3). We then focused on let-7f because it was the most downregulated fecal miRNA, and because its production could be directly correlated with the lack of intestinal γδ T cells in γδ^−/−^ mice whereas miR-876-5p, which was upregulated, was likely produced by other intestinal cells to compensate for the reduced levels of γδ T cell-produced let-7f. We then sorted γδ T cells from WT mice and confirmed by RT-qPCR that let-7f is expressed in γδ T cells from both LP and IEL compartments (Fig. 7c). Of note, we also found that LP and IEL ab T cells expressed let-7f (Supplemental Fig. 6e), but the significant reduction of let-7f in the feces of γδ^−/−^ mice suggest that γδ T cells play an important role in secreting this miRNA.

We then investigated *R. gnavus* gene targets of let-7f by blasting the sequence of let-7f against the whole genome of *R. gnavus*. As shown in Fig. 7d, we found that two bacterial genes for *R. gnavus* were predicted to be targeted by let-7: FXV78_05965, a putative Hsp20/alpha crystallin family protein, which are chaperones to protect other proteins against stress-induced denaturation and aggregation [47], and BN481_02546, the phosphotransferase system EIIC, responsible for selectively transporting sugar molecules across the inner bacterial membrane [48]. To investigate whether let-7f promoted bacterial growth in vivo, we orally administered 1000 pmol/mouse of let-7f or its scrambled sequence for 7 days and measured *R. gnavus* by RT-qPCR in fecal samples collected 1 day after the last dose. Of note, we have previously shown that 1000 pmol is the optimal miRNA dose for in vivo experiments [13]. We found that let-7f, but not the scrambled sequence, promoted *R. gnavus* growth (Fig. 7e), confirming that γδ T cell-derived let-7f can select for specific commensals. Importantly, we also found that let-7f is predicted to target two bacterial genes for *Akkermansia muciniphila* (Supplemental Fig. 6f), another mucus-degrading microbe [42] that we found to be reduced in γδ^−/−^ microbiota (Supplemental Fig. 3d). Accordingly, oral administration of let-7f to γδ^−/−^ mice increased the abundance of *A. muciniphila* in these mice (Supplemental Fig. 6g). Notably, we have previously found that *A. muciniphila* have intestinal regulatory functions in a mouse model of multiple sclerosis [13].

We next investigated whether oral administration of let-7f restored oral tolerance in γδ^−/−^ mice. To address this, WT and γδ^−/−^ mice were treated with 1000 pmol of let-7f or its scrambled sequence for 7 days and 1 day after the last dose, OVA was provided in the drinking water for 5 days. We then immunized mice with OVA/CFA and 10 days after immunization, spleens were collected for proliferation assay (Fig. 7f). We found that γδ^−/−^ mice treated with let-7f, but not its scrambled sequence, developed oral tolerance, as indicated by the reduction of splenocyte proliferation against OVA (Fig. 7g). Let-7f did not enhance or impair oral tolerance in WT mice. Furthermore, oral administration of let-7f restored Th17, but not Treg cell levels (Fig. 7h, i), suggesting that increased Th17 rather than decreased Treg cells is responsible for impaired oral tolerance induction in γδ^−/−^ mice. Thus, oral administration of let-7f promotes *R. gnavus* growth and rescues mucosal tolerance in γδ^−/−^ mice.

## Discussion

It has long been recognized that γδ T cells play a critical role in mucosal tolerance [29, 30, 49, 50], though the mechanism(s) by which they exert this effect is unknown. It was subsequently shown that the gut microbiota also plays an important role in mucosal tolerance as oral tolerance is defective in germ-free mice or in animals treated with antibiotics [21, 51, 52]. Because it is known that γδ T cells interact with the gut microbiota [53, 54], we asked whether γδ T cells promoted mucosal tolerance and intestinal homeostasis via the microbiome. We demonstrate that γδ T cells regulate the microbiota, which in turn maintains γδ T cells in the gut. This crosstalk was particularly relevant for the small intestine as we did not find major changes in the colon. It has been demonstrated that γδ T cells from the IEL compartment are not affected by the gut microbiome [9, 55] and we found that both IEL and LP γδ T cells significantly decreased after antibiotic treatment. Importantly, those studies were conducted in GF mice, which are broadly impaired in many aspects of development and early immune education [56]. We believe that this discrepancy is related to the fact that in specific pathogen-free (SPF) conditions, intestinal γδ T cells develop along with the microbiota forming intricate interactions, which when disrupted by an acute broad-spectrum antibiotic treatment, results in a transient reduction of these cells.

We found that the SILP of γδ^−/−^ mice had increased frequencies of Th17 cells and decreased frequencies of Treg cells. This Th17/Treg cell imbalance was likely caused by the reduced production of IL-10 by CX3CR1^+^ MNPs, which favors Treg cell activation and survival and suppressed Th17 cell-induced inflammation [24–27], rather than by the impaired migration of cDC1s to the mLN, which is critical for Treg cell differentiation [37]. This is because we found that frequencies of OVA-specific Treg cells were increased in the mLN of γδ^−/−^ mice regardless of OVA feeding, but these cells were reduced in the SILP of γδ^−/−^ mice and did not increase following OVA administration. However, our findings do not rule out a possible defect in the gut homing imprint of Treg cells in the mLN and their subsequent migration to the SILP, which is also controlled by cDC1s [37]. Importantly, colonization of γδ^−/−^ mice with WT microbiota or with the γδ^−/−^ mouse depleted microbe *R. gnavus*, restored the migratory properties of cDC1s and the IL-10 production by CX3CR1^+^ MNPs, normalized Th17/Treg cell frequencies to WT levels, and rescued oral tolerance, confirming that γδ T cells play a critical role in shaping the gut microbiota to maintain a tolerogenic milieu in the gut.

Noteworthy is the fact that transfer of γδ^−/−^ microbiota into WT mice, which are sufficient for γδ T cells, led to defective oral tolerance induction that was associated with reduced Treg and increased Th17 cells in the LP. We believe that this occurred because of the timing that mice were exposed to the microbiota and antigen (OVA). Mice received OVA for tolerance induction 3 days after microbiota transfer. At that time, γδ T cells in WT mice are likely responding to the new microbial environment by secreting AMPs and let-7f. However, oral tolerance induction 3 days after microbiota transfer does not provide sufficient time for γδ T cells to restructure the altered microbiota. It is possible that at a later time point the microbiota recovers, resulting in the rescue of the phenotype.

We then sequenced γδ^−/−^ microbial 16S rRNA and found a number of bacteria that were either increased or decreased and thus candidates for playing a role in mucosal tolerance. We focused on *R. gnavus* and *P. excrementihominis* as these microbes were altered in different cohorts of mice as well as in our studies of oral tolerance using EAE (not shown). Thus, we first colonized γδ^−/−^ mice with *R. gnavus* and found that oral tolerance was restored in these mice, and this was associated with the normalization of cDC1 migration to the mLN and IL-10 production by CX3CR1^+^ MNPs, increased Tregs, and decreased Th17 cells in the SILP of γδ^−/−^ mice. Consistent with this, *R. gnavus* has been correlated with Treg cell differentiation in mice [57, 58]. We then colonized WT mice with *P. excrementihominis* but did not find that *P. excrementihominis* impaired oral tolerance. Altogether, these findings indicate that *R. gnavus* favors an intestinal homeostasis, which is critical for oral tolerance induction.

Interactions between the gut microbiota and the intestinal epithelium are crucial for intestinal homeostasis. In this regard, Kim and colleagues have shown that CX3CR1^+^ MNPs, via secretion of IL-10, promote the generation of Treg cells in response to food antigens, which favors oral tolerance induction. Disruption of the gut microbiota by antibiotics affects this immune regulatory loop and impairs oral tolerance induction whereas colonization of antibiotic-treated mice with microbes that adhere to the intestinal epithelium normalizes levels of IL-10-producing CX3CR1^+^ MNPs and rescues oral tolerance [26]. This suggests that intestinal epithelial barrier is critical for oral tolerance and that microbial adherence plays an important role in gut homeostasis. A crucial factor that regulates microbial adherence to the gut epithelium is the mucus. A thick mucus layer affects the interaction between microbes and intestinal epithelial cells [44]. We found that γδ^−/−^ mice were deficient in *R. gnavus* and *Akkermansia muciniphila*, which are microbes that degrade mucus [40–42]. Consistent with this, we found that γδ^−/−^ mice have a thicker mucus layer in the small intestine as compared to WT mice and that colonization of γδ^−/−^ mice with *R. gnavus* decreased mucus layer thickness. Moreover, *R. gnavus* restored IL-10 production by CX3CR1^+^ MNPs. Taken together, an increased mucus layer in γδ^−/−^ mice may be associated with reduced microbial adherence to the epithelium, which in turn decreases IL-10 production by CX3CR1^+^ MNPs, affects Treg/Th17 balance in the gut, and impairs oral tolerance.

We have identified miRNAs in the feces and found that they play a role in modulating the gut microbiota [13, 46]. These miRNAs can enter bacteria, regulate bacterial gene transcripts, and affect bacterial growth. For example, we found that miR-30d regulates the expression of a newly identified lactase in *Akkermansia muciniphila* and increases the abundance of *Akkermansia* in the gut. The expanded *Akkermansia* in turn increases Tregs and ameliorates EAE [13]. In the current study, we found that intestinal γδ T cells produce let-7f, a miRNA that is highly conserved across species [59]. Although the mechanisms by which γδ T cells are stimulated to secrete miRNAs are not known, it may relate to the ability of γδ T cells to respond to microbes or microbial metabolites directly via pathogen recognition receptors (PRRs) including toll-like receptors [60], or indirectly to signals from the intestinal epithelia following epithelial cell activation by microbes [53, 54]. We found that restoring let-7f levels in γδ^−/−^ mice by oral gavage promoted *R. gnavus* (and *A. muciniphila*) growth and rescued mucosal tolerance, demonstrating a critical role for miRNAs in regulating microbial homeostasis in the gut. Notably, we found that ab T cells from both IEL and LP compartments also produced let-7f. Moreover, we have previously shown that let-7f is significantly reduced in the feces of mice in which Dicer, a critical protein involved in miRNA synthesis, is specifically deleted in the gut epithelium, indicating that epithelial cells are also a source of fecal let-7f [46]. This is not surprising since the let-7 family of miRNAs plays ubiquitous roles in distinct cell types involved in several functions [61]. However, the fact that in the absence of γδ T cells (γδ^−/−^ mice) both ab T and epithelial cells are still functional, we concluded that let-7f produced by intestinal gd T cells play a critical role in promoting the growth of *R. gnavus* and restoring oral tolerance and that intestinal γδ T cells are a major source of let-7f. However, further experiments are necessary to determine whether the effects of let-7f on oral tolerance is due to direct secretion of let-7f by γδ T cells or if γδ T cells indirectly contribute to the production of let-7f by another cell type in the mucosa.

Importantly, the let-7 family of miRNAs has been shown to negatively regulate proliferation, differentiation, and chemokine-mediated migration of pathogenic Th17 cells to the CNS [62]. Thus, let-7f may have a dual effect in promoting mucosal tolerance by inducing the growth of tolerogenic microbes and by modulating Th17 cells. Consistent with this, oral administration of let-7f restored Th17, but not Treg cell levels in the SILP of γδ^−/−^ mice. It is also possible that other decreased fecal miRNAs we found in γδ^−/−^ mouse feces (miR-762, miR-135a and miR-876-5p) may be involved in the normalization of Treg cell frequencies in the SILP of γδ^−/−^ mice. This is consistent with our microbiota swap experiments in which transfer of WT microbiota, which contains not only live microbes including *R. gnavus*, but also fecal miRNAs, normalized both Th17 and Treg cells.

## Conclusions

In summary, we have found that a dysbiotic gut microbiota is a major factor that explains the defective mucosal tolerance that has been observed in γδ^−/−^ mice. Furthermore, we found that intestinal γδ T cells secrete let-7f that promotes the growth of *R. gnavus* to maintain a homeostatic microbiota. A healthy microbiota induces migration of cDC1s to the mLN and the production of IL-10 by CX3CR1^+^ MNPs, which in turn favors Treg cell differentiation, activation, and survival and decreases Th17 cell induction in the gut. This leads to a tolerogenic intestinal milieu that contributes to mucosal tolerance development. Moreover, it is possible that oral administration of let-7f could be used therapeutically to modulate the gut microbiota and correct intestinal dysbiosis that interferes with oral tolerance induction in patients with autoimmune and inflammatory diseases.

## Supplementary Information

**Additional file 1: Supplemental Figure 1.** Antibiotic specific effects on oral tolerance. (a) Microbiota was depleted with a combination of 4 antibiotics (QUAD) in the drinking water for 3 days, and control mice did not receive antibiotics (*n*=10 mice each). Two days later, half of each group were then fed OVA in the drinking water for 5 days. Antibiotics and OVA were stopped and two days later mice were immunized with OVA/CFA. **(b)** Responsiveness to OVA was measured by delayed type hypersensitivity by injecting OVA into the footpad 21 days after OVA/CFA immunization. Data are mean + SEM; *n*=5 mice/group; one-way ANOVA. **(c)** Principal coordinate analysis of unweighted UniFrac distances during OVA feeding and antibiotic treatment for days -9 through +1. **(d)** Fecal microbiota composition over 24 days. Bars represent an average of relative abundance of *n*=5 mice within each treatment group. **Supplemental Figure 2.** Cytokine expression in CD4 and CD8 T cells from the lamina propria of WT vs. γδ^-/-^ mice. **(a-d)** Small intestine lamina propria of naïve WT and γδ^-/-^ mice were collected and flow cytometric analysis performed for IL-10 **(a)** and IFN-γ **(b)** in CD4 T cells, and IFN-γ **(c)** and IL-17A **(d)** in CD8 T cells. Data are mean + SEM; *n*=4 mice/group; Student’s t-test. * *p* < 0.05. Results are representative of at least two independent experiments. **Supplemental Figure 3.** Microbiota alterations associated with the loss of oral tolerance. **(a)** Microbiota was depleted with a combination of 4 antibiotics (ABX) in the drinking water for 3 days and one day later, microbiota from WT and γδ^-/-^ mice were swapped. Microbiota differences were characterized 17 days post colonization by 16S rRNA sequencing of the 16S rRNA gene. **(b)** Principal coordinates analysis of unweighted UniFrac distances. **(c)** ADONIS testing of unweighted UniFrac distances shows that composition varies by microbiota donor source (23.1%), genotype (8.50%), and anatomical location (24.7%), *p* < 0.001, ADONIS test. **(d)** Bacteria elevated in γδ^-/-^ microbiota colonized mice (green) or WT microbiota colonized mice (black). Linear discriminant analysis (LEfSe) with microbiota source as set as the class and recipient genotype set as the subclass. **(e)** Relative abundance of select taxa associated with γδ^-/-^ microbiota vs WT microbiota colonization. **Supplemental Figure 4.** γδ^-/-^ microbiota characterization and enriched pathways. **(a)** Fecal microbiota composition of WT vs. γδ ^-/-^ mice in the small (SI) and large (LI) intestines. **(b)** KEGG pathways enriched in the WT microbiota. **(c)** KEGG pathways enriched in the γδ^-/-^ microbiota. **(d)** Examples of altered KEGG pathways in WT and γδ^-/-^ microbiota in the SI and LI. Bars represent mean + SEM; * *p* < 0.05; LEfSe. **(e-f)** Oral tolerance is not impaired by *P. excrementihominis* colonization. **(e)** WT and γδ^-/-^ mice were gavaged with *P. excrementihominis* once a week for 4 weeks. One week later, half of each group were fed OVA in the drinking water for 5 days. OVA continuous feeding was stopped and two days later mice were immunized with OVA/CFA. **(f)** Responsiveness to OVA was measured by splenocyte proliferation upon 100 µg/ml of OVA stimulation. Data are mean + SEM; *n*=5 mice/group; one-way ANOVA. **(g)** Relative abundance of *P. excrementihominis* in WT and γδ^-/-^ mice colonized with *P. excrementihominis*. Data are mean + SEM; *n*=10 mice/group; one-way ANOVA. NS=non-significant, * *p* < 0.05. Results are representative of at least two independent experiments. **Supplemental Figure 5.** γδ^-/-^ mice have a thicker intestinal mucus layer. **(a)** Representative images of the jejunum from WT and γδ^-/-^ mice colonized or not with *R. gnavus* (Rg) as describe in Fig. 6. 5-μm serial sections were stained with PAS for mucus analysis. Magnification of 20x. Scale bars, 250 mm. Percentage of PAS per mm^2^ of tissue were calculated using FIJI as described in the Methodology section. **(b)** Bar graphs showing mucus layer thickness in the duodenum (Duo), jejunum (Jej), and ileum (IL) from WT and γδ^-/-^ mice colonized or not with *R. gnavus*. Data are mean + SEM; *n*=5-9 mice/group; one-way ANOVA. * *p* < 0.05. **(c)** Representative images of the duodenum, jejunum, and ileum from WT and γδ^-/-^ mice colonized or not with *R. gnavus* (Rg). 20-μm serial sections were stained with AlexaFluor-488-conjugated-wheat germ agglutinin. Magnification of 10x. Scale bars, 500 µm. Results are representative of at least two independent experiments. **Supplemental Figure 6.** γδ^-/-^ microbiota induces antimicrobial peptide production by γδ T cells and let-7f-induced *A. muciniphila* growth. **(a-c)** RT-qPCR analysis of mRNA expression levels of *Reg3a*, *Reg3b* and *Reg3g* of sorted αβ T cells from small intestine (SI) intraepithelial lymphocyte (IEL, **a**) and lamina propria (SILP, **b**) compartments and sorted epithelial cells from the small intestine (c) of WT and γδ^-/-^ mice at steady state. **(d)** Microbiota was depleted in WT recipient mice with a combination of 4 antibiotics in the drinking water for 3 days and one day later they were colonized with either WT or γδ^-/-^ microbiota. Mice were euthanized 2 weeks later, and ab and γδ T cells from SI-IEL layer and SILP sorted for RT-qPCR analysis of *Reg3a*, *Reg3b* and *Reg3g* mRNA expression. Data are mean + SEM; *n*=5-6 mice/group; one-way ANOVA. **(e)** IEL and LP αβ T cells were sorted and let-7f expression measured by RT-qPCR. Relative expression to miR-21 (the most abundant fecal miRNA) is shown. Data are mean + SEM; *n*=4 mice pooled into 2 samples. **(f)** Bacterial genes of *A. muciniphila* that are predicted to be targeted by let-7f, indicated by sequence blast and predicted for secondary structure property (minimum free energy, mfe) by RNAhybrid. **(g)** Let-7f or its scrambled sequence (Scr) were orally given to WT and γδ^-/-^ mice for 7 consecutive days at a dose of 1000 pmol/mouse/day. On the last day of miRNA administration, fecal samples from WT and γδ^-/-^ mice were collected and RT-qPCR performed to detect *A. muciniphila* levels. Data are mean + SEM; *n*=5 mice/group; Student’s t-test. ND= not detected, NS=non-significant, * *p* < 0.05, **** *p*<0.0001.

**Additional file 2: Supplementary Table 1.** The microbiota promotes the induction of oral tolerance and maintains γδ T cells.

**Additional file 3: Supplementary Table 2.** γδ^-/-^microbiota induces intestinal inflammation in WT mice.

**Additional file 4: Supplementary Table 3.** Intestinal γδ T cells secrete miRNAs to promote rhe growth of *Ruminococcus gnavus.*
