# Supplementary material for: Gamma-delta T cells modulate the microbiota and fecal micro-RNAs to maintain mucosal tolerance
Source: Microbiome. 2023 Feb 23;11:32. doi: 10.1186/s40168-023-01478-1 (PMC9948450; doi:10.1186/s40168-023-01478-1)
Supplement: Supplementary file 2 — Additional file 1: Supplemental Figure 1. Antibiotic specific effects on oral tolerance. (a) Microbiota was depleted with a combination of 4 antibiotics (QUAD) in the drinking water for 3 days, and control mice did not receive antibiotics (n=10 mice each). Two days later, half of each group were then fed OVA in the drinking water for 5 days. Antibiotics and OVA were stopped and two days later mice were immunized with OVA/CFA. (b) Responsiveness to OVA was measured by delayed type hypersensitivity by injecting OVA into the footpad 21 days after OVA/CFA immunization. Data are mean + SEM; n=5 mice/group; one-way ANOVA. (c) Principal coordinate analysis of unweighted UniFrac distances during OVA feeding and antibiotic treatment for days -9 through +1. (d) Fecal microbiota composition over 24 days. Bars represent an average of relative abundance of n=5 mice within each treatment group. Supplemental Figure 2. Cytokine expression in CD4 and CD8 T cells from the lamina propria of WT vs. γδ-/- mice. (a-d) Small intestine lamina propria of naïve WT and γδ-/- mice were collected and flow cytometric analysis performed for IL-10 (a) and IFN-γ (b) in CD4 T cells, and IFN-γ (c) and IL-17A (d) in CD8 T cells. Data are mean + SEM; n=4 mice/group; Student’s t-test. * p < 0.05. Results are representative of at least two independent experiments. Supplemental Figure 3. Microbiota alterations associated with the loss of oral tolerance. (a) Microbiota was depleted with a combination of 4 antibiotics (ABX) in the drinking water for 3 days and one day later, microbiota from WT and γδ-/- mice were swapped. Microbiota differences were characterized 17 days post colonization by 16S rRNA sequencing of the 16S rRNA gene. (b) Principal coordinates analysis of unweighted UniFrac distances. (c) ADONIS testing of unweighted UniFrac distances shows that composition varies by microbiota donor source (23.1%), genotype (8.50%), and anatomical location (24.7%), p < 0.001, ADONIS test. (d) Bacteria elevat [file 40168_2023_1478_MOESM1_ESM.docx]

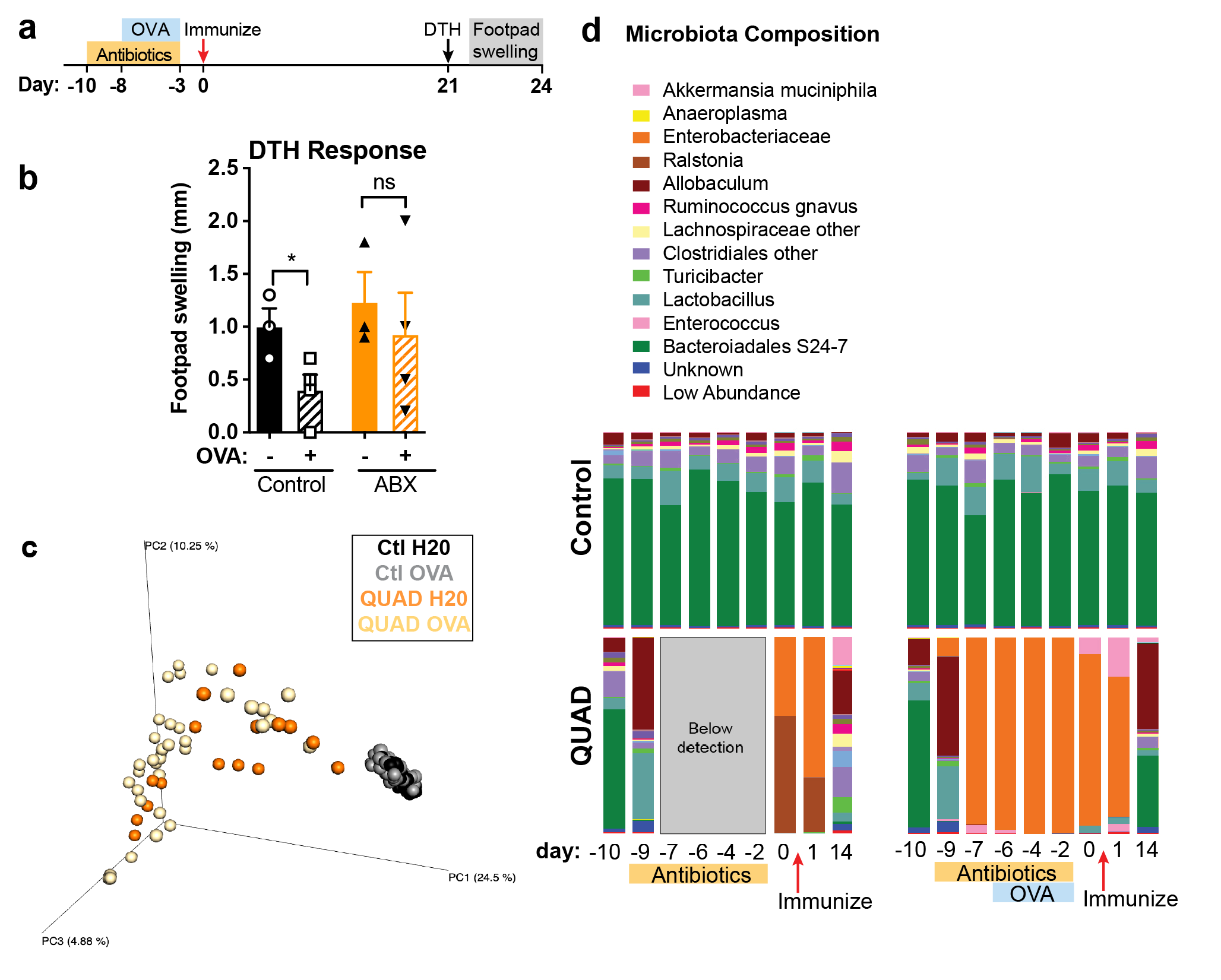


**Supplemental Figure 1. Antibiotic specific effects on oral tolerance. (a)** Microbiota was depleted with a combination of 4 antibiotics (QUAD) in the drinking water for 3 days, and control mice did not receive antibiotics (n=10 mice each). Two days later, half of each group were then fed OVA in the drinking water for 5 days. Antibiotics and OVA were stopped and two days later mice were immunized with OVA/CFA. **(b)** Responsiveness to OVA was measured by delayed type hypersensitivity by injecting OVA into the footpad 21 days after OVA/CFA immunization. Data are mean + SEM; n=5 mice/group; one-way ANOVA. **(c)** Principal coordinate analysis of unweighted UniFrac distances during OVA feeding and antibiotic treatment for days -9 through +1. **(d)** Fecal microbiota composition over 24 days. Bars represent an average of relative abundance of n=5 mice within each treatment group.


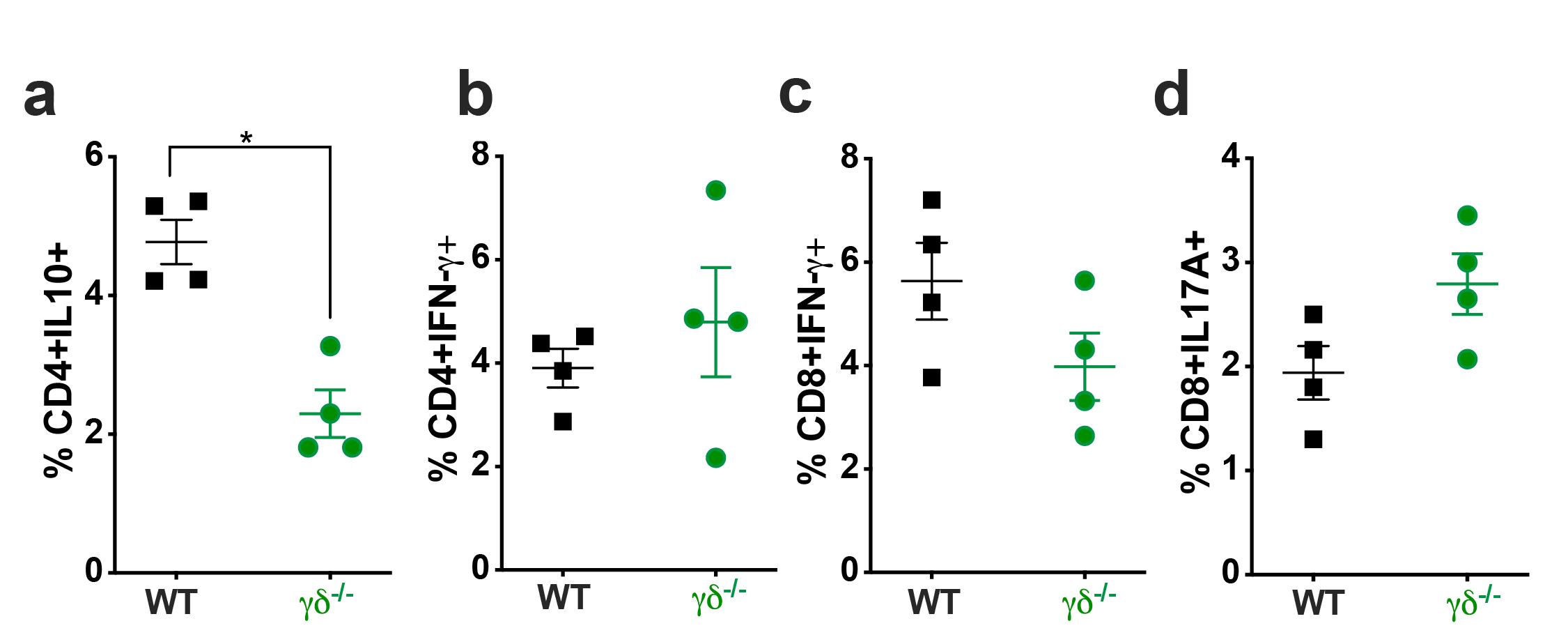


**Supplemental Figure 2. Cytokine expression in CD4 and CD8 T cells from the lamina propria of WT vs. γδ^-/-^ mice. (a-d)** Small intestine lamina propria of naïve WT and γδ^-/-^ mice were collected and flow cytometric analysis performed for IL-10 **(a)** and IFN-γ **(b)** in CD4 T cells, and IFN-γ **(c)** and IL-17A **(d)** in CD8 T cells. Data are mean + SEM; n=4 mice/group; Student’s t-test. * p < 0.05. Results are representative of at least two independent experiments.


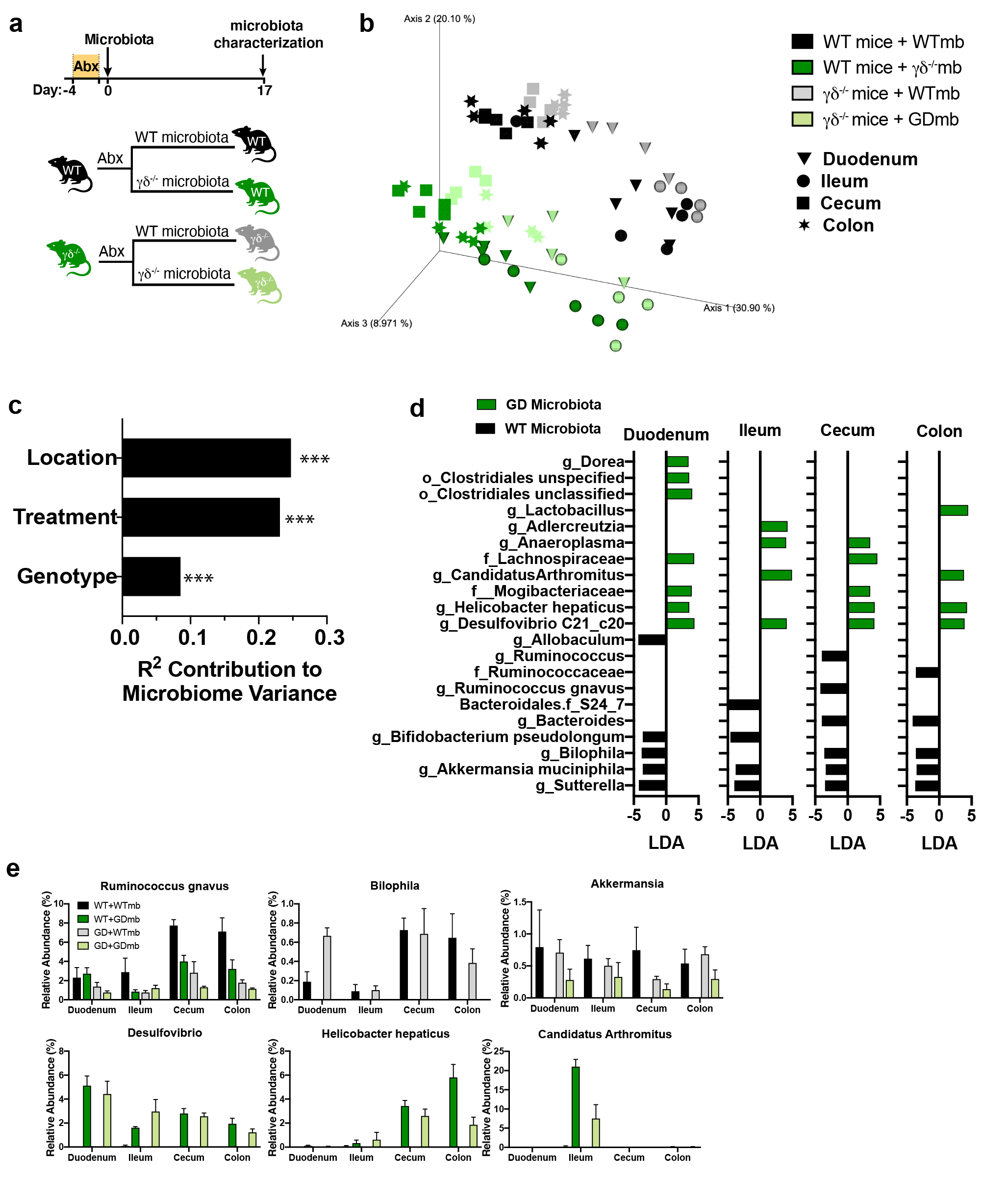


**Supplemental Figure 3. Microbiota alterations associated with the loss of oral tolerance.** **(a)** Microbiota was depleted with a combination of 4 antibiotics (ABX) in the drinking water for 3 days and one day later, microbiota from WT and γδ^-/-^ mice were swapped. Microbiota differences were characterized 17 days post colonization by 16S rRNA sequencing of the 16S rRNA gene. **(b)** Principal coordinates analysis of unweighted UniFrac distances. **(c)** ADONIS testing of unweighted UniFrac distances shows that composition varies by microbiota donor source (23.1%), genotype (8.50%), and anatomical location (24.7%), p < 0.001, ADONIS test. **(d)** Bacteria elevated in γδ^-/-^ microbiota colonized mice (green) or WT microbiota colonized mice (black). Linear discriminant analysis (LEfSe) with microbiota source as set as the class and recipient genotype set as the subclass. **(e)** Relative abundance of select taxa associated with γδ^-/-^ microbiota vs WT microbiota colonization.

**
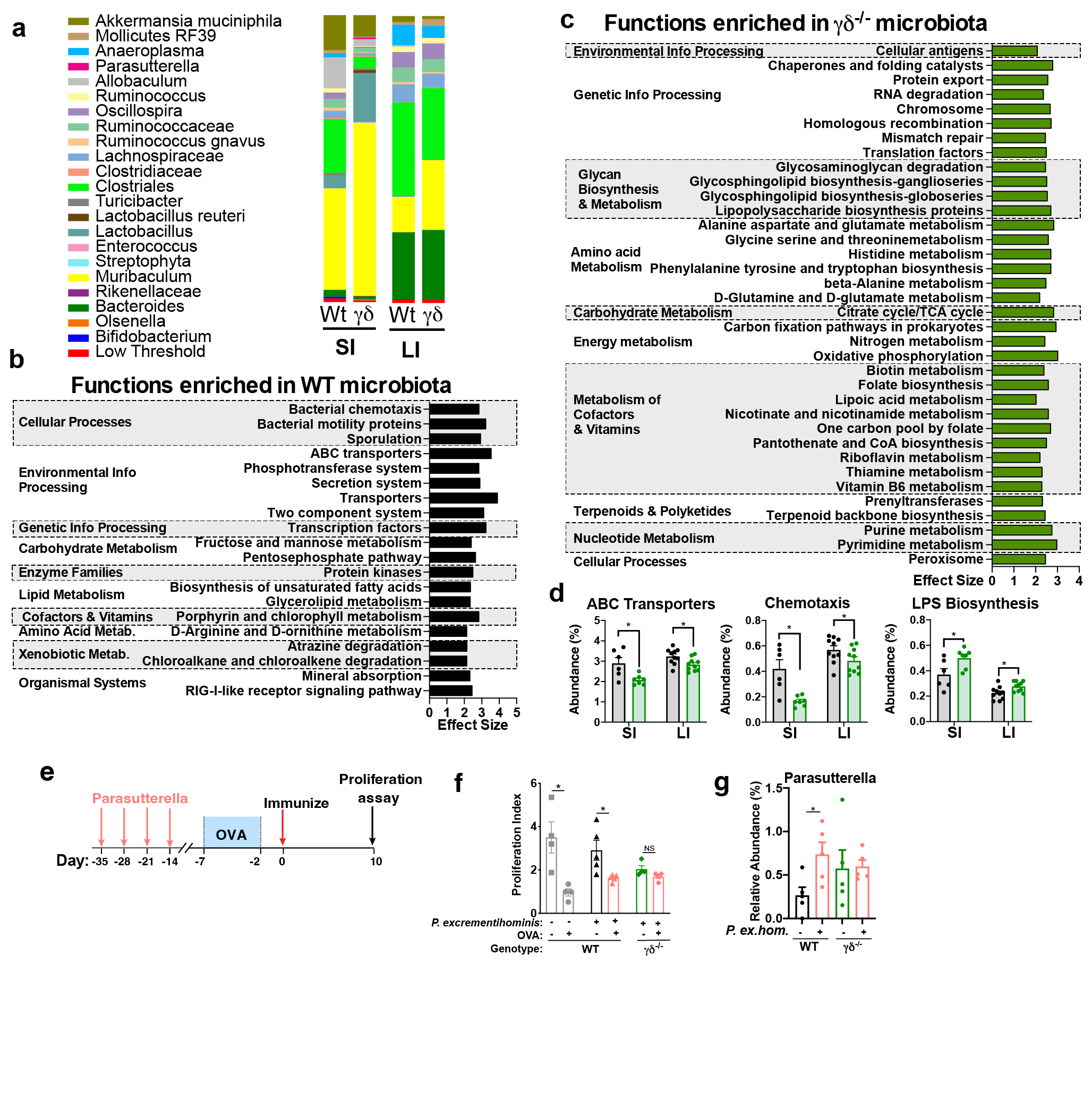
**

**Supplemental Figure 4. γδ^-/-^ microbiota characterization and enriched pathways. (a)** Fecal microbiota composition of WT vs. γδ^-/-^ mice in the small (SI) and large (LI) intestines. **(b)** KEGG pathways enriched in the WT microbiota. **(c)** KEGG pathways enriched in the γδ^-/-^ microbiota. **(d)** Examples of altered KEGG pathways in WT and γδ^-/-^ microbiota in the SI and LI. Bars represent mean + SEM; * p < 0.05; LEfSe. **(e-f)** Oral tolerance is not impaired by *P. excrementihominis* colonization. **(e)** WT and γδ^-/-^ mice were gavaged with *P. excrementihominis* once a week for 4 weeks. One week later, half of each group were fed OVA in the drinking water for 5 days. OVA continuous feeding was stopped and two days later mice were immunized with OVA/CFA. **(f)** Responsiveness to OVA was measured by splenocyte proliferation upon 100 μg/ml of OVA stimulation. Data are mean + SEM; n=5 mice/group; one-way ANOVA. **(g)** Relative abundance of *P. excrementihominis* in WT and γδ^-/-^ mice colonized with *P. excrementihominis*. Data are mean + SEM; n=10 mice/group; one-way ANOVA. NS=non-significant, * p < 0.05. Results are representative of at least two independent experiments.

**Supplemental Figure 5. γδ^-/-^ mice have a thicker intestinal mucus layer. (a)** Representative images of the jejunum from WT and γδ^-/-^ mice colonized or not with *R. gnavus* (Rg) as describe in Fig. 6. 5-μm serial sections were stained with PAS for mucus analysis. Magnification of 20x. Scale bars, 250 μm. Percentage of PAS per μm^2^ of tissue were calculated using FIJI as described in the Methodology section. **(b)** Bar graphs showing mucus layer thickness in the duodenum (Duo), jejunum (Jej), and ileum (IL) from WT and γδ^-/-^ mice colonized or not with *R. gnavus*. Data are mean + SEM; n=5-9 mice/group; one-way ANOVA. * p < 0.05. **(c)** Representative images of the duodenum, jejunum, and ileum from WT and γδ^-/-^ mice colonized or not with *R. gnavus* (Rg). 20-μm serial sections were stained with AlexaFluor-488-conjugated-wheat germ agglutinin. Magnification of 10x. Scale bars, 500 μm. Results are representative of at least two independent experiments.

**Supplemental Figure 6. γδ^-/-^ microbiota induces antimicrobial peptide production by γδ T cells and let-7f-induced *A. muciniphila* growth. (a-c)** RT-qPCR analysis of mRNA expression levels of *RegIIIa*, *RegIIIb* and *RegIIIg* of sorted αβ T cells from small intestine (SI) intraepithelial lymphocyte (IEL, **a**) and lamina propria (SILP, **b**) compartments and sorted epithelial cells from the small intestine (c) of WT and γδ^-/-^ mice at steady state. **(d)** Microbiota was depleted in WT recipient mice with a combination of 4 antibiotics in the drinking water for 3 days and one day later they were colonized with either WT or γδ^-/-^ microbiota. Mice were euthanized 2 weeks later, and αβ and γδ T cells from SI-IEL layer and SILP sorted for RT-qPCR analysis of *RegIIIa*, *RegIIIb* and *RegIIIg* mRNA expression. Data are mean + SEM; n=5-6 mice/group; one-way ANOVA. **(e)** IEL and LP αβ T cells were sorted and let-7f expression measured by RT-qPCR. Relative expression to miR-21 (the most abundant fecal miRNA) is shown. Data are mean + SEM; n=4 mice pooled into 2 samples. **(f)** Bacterial genes of *A. muciniphila* that are predicted to be targeted by let-7f, indicated by sequence blast and predicted for secondary structure property (minimum free energy, mfe) by RNAhybrid. **(g)** Let-7f or its scrambled sequence (Scr) were orally given to WT and γδ^-/-^ mice for 7 consecutive days at a dose of 1000 pmol/mouse/day. On the last day of miRNA administration, fecal samples from WT and γδ^-/-^ mice were collected and RT-qPCR performed to detect *A. muciniphila* levels. Data are mean + SEM; n=5 mice/group; Student’s t-test. ND= not detected, NS=non-significant, * p < 0.05, **** p<0.0001.
